# Genome-Wide Transcriptional Response of Silkworm (*Bombyx mori)* to Infection by the Microsporidian *Nosema bombycis*

**DOI:** 10.1371/journal.pone.0084137

**Published:** 2013-12-30

**Authors:** Zhengang Ma, Chunfeng Li, Guoqing Pan, Zhihong Li, Bing Han, Jinshan Xu, Xiqian Lan, Jie Chen, Donglin Yang, Quanmei Chen, Qi Sang, Xiaocun Ji, Tian Li, Mengxian Long, Zeyang Zhou

**Affiliations:** 1 The State Key Laboratory of Silkworm Genome Biology, Southwest University, Chongqing, China; 2 College of Life Sciences, Chongqing Normal University, Chongqing, China; 3 Key Laboratory for Sericulture Functional Genomics and Biotechnology of Agricultural Ministry, Southwest University, Chongqing, China; University of Ottawa, Canada

## Abstract

Microsporidia have attracted much attention because they infect a variety of species ranging from protists to mammals, including immunocompromised patients with AIDS or cancer. Aside from the study on *Nosema ceranae*, few works have focused on elucidating the mechanism in host response to microsporidia infection. *Nosema bombycis* is a pathogen of silkworm pébrine that causes great economic losses to the silkworm industry. Detailed understanding of the host (*Bombyx mori*) response to infection by *N. bombycis* is helpful for prevention of this disease. A genome-wide survey of the gene expression profile at 2, 4, 6 and 8 days post-infection by *N. bombycis* was performed and results showed that 64, 244, 1,328, 1,887 genes were induced, respectively. Up to 124 genes, which are involved in basal metabolism pathways, were modulated. Notably, *B. mori* genes that play a role in juvenile hormone synthesis and metabolism pathways were induced, suggesting that the host may accumulate JH as a response to infection. Interestingly, *N. bombycis* can inhibit the silkworm serine protease cascade melanization pathway in hemolymph, which may be due to the secretion of serpins in the microsporidia. *N. bombycis* also induced up-regulation of several cellular immune factors, in which CTL11 has been suggested to be involved in both spore recognition and immune signal transduction. Microarray and real-time PCR analysis indicated the activation of silkworm Toll and JAK/STAT pathways. The notable up-regulation of antimicrobial peptides, including gloverins, lebocins and moricins, strongly indicated that antimicrobial peptide defense mechanisms were triggered to resist the invasive microsporidia. An analysis of *N. bombycis-*specific response factors suggested their important roles in anti-microsporidia defense. Overall, this study primarily provides insight into the potential molecular mechanisms for the host-parasite interaction between *B. mori* and *N. bombycis* and may provide a foundation for further work on host-parasite interaction between insects and microsporidia.

## Introduction

As a group of obligate intracellular single-cell spore-forming organisms, microsporidia can infect a variety of hosts ranging from protists to mammals. However, almost half of the reported genera of microsporidia use insects as primary hosts, and microsporidia infection usually has chronic and sublethal effects on hosts [1]. The infection by microsporidia can be disastrous to economic insects such as silkworms and honeybees, primarily due to horizontal and vertical transmission. Thus, leading to enormous loss in relevant industries. In addition, microsporidiosis, which is caused by microsporidia, has been recognized in different groups of people including patients with AIDS and cancer, organ transplant recipients, diabetics, travelers, children, and the elderly [2]. Microsporidiosis is a threat to human health. Microsporidia have a highly specialized invasion organelle, the polar tube [3], [4]. The polar tubes of active spores can extrude and penetrate the plasma membrane of host cells and transfer infectious protoplasm into the cells followed by spore proliferation [1], [5], [6]. Polar tubes mainly consist of three polar tube proteins (PTP1, PTP2 and PTP3) that interact with each other [7]. During infection, the dense and rigid spore wall can prevent microsporidian from host attack [8]. Spore wall proteins have been reported to mediate the ejection of polar tubes by adjusting their permeability and may play an important role in the pathogenic infection process for spore adherence to cell lines [9], [10]. Recently, subtilisin-like serine proteases (SLPs), considered to be potential virulence factors, have been implicated in the polar tube extrusion process [11].

Although many studies of the infection mechanism of microsporidian have been reported, few studies have focused on elucidating the mechanism of the host response to microsporidia infection. Thus, investigation on the interplay of genome-wide expression profile of hosts and parasites is critical for understanding the mechanisms of self-protection, resistance and defense against invasive microsporidia. Microarray technology can be used to monitor gene expression profiles on a whole-genome scale using a single chip to assess the expression of thousands of genes simultaneously. This technique is a powerful tool for identifying genes that participate in the host response to parasite infection [12]. Recently, Rosenblum EB used microarray to reveal that *Silurana tropicalis* had a strong physiological effect (i.e., decreased expression of a large number of cytochrome p450 family proteins and upregulation of the expression level of heat shock proteins and genes associated with cellular integrity) and weak immune response after infection with the chytrid fungus *Batrachochytrium dendrobatidis* [13]. Similarly, the significant up-regulation of glycoprotein metabolism in human ileocecal epithelial cells (HCT-8) after infection with *Cryptosporidium parvum* was examined [14]. In honeybee, the gut response to *Nosema ceranae* infection was investigated using NimbelGen HD2 tiling microarrays and demonstrated that the immune response of the bee responded to *N. ceranae* infection by increasing oxidative stress [15]. The genomic sequences for both silkworm *Bombyx mori* and microsporidia *Nosema bombycis* are readily available, they are a good model system for investigating the interplay between host defense and the microsporidia [16], [17]. In addition, the accomplishment of the 23K Silkworm Genome Array also makes it more convenient to obtain a genome-wide transcriptional profile of the silkworm [18]. Subsequently, a survey using the silkworm genome-wide microarray for host response of silkworm *B. mori* to *Bacillus bombyseptieus* (*Bb*) was reported, and it showed that *Bb* can cause melanization during the early stage of infection and trigger the host immune response [19].

To investigate the silkworm response to infection by *N. bombycis*, we performed microarray analysis of infected and uninfected silkworms at 2, 4, 6 and 8 days post-infection (dpi). Our results revealed that a strong and complex host response is induced by *N. bombycis* infection, and our study provides a comprehensive transcriptional profile of the interaction between microsporidia and its host.

## Materials and Methods

### Silkworm Strain and Preparation of Artificial Diet

The silkworm strain, *Dazao*, used in this study was provided by the Silkworm Gene Resources of Southwest University, Chongqing, China. The artificial diet was purchased from Nichiku Yakuhin Kogyo Corporation (Japan). About 100 grams of artificial diet and 280 ml of ddH_2_O were stirred well in an iron box and then steamed at 98°C for 25 min. The steamed artificial diet stored at 4°C for feeding. The silkworms were reared on an artificial diet at 25°C and maintained at a suitable humidity of approximately 70% together with a photoperiod of 12 h of light and 12 h of dark up to the 2nd molting for infection experiments.

### *N. bombycis* Spore Purification and Morphological Observations

The *N. bombycis* isolate CQ1 was obtained from infected silkworms in Chongqing, China, and was conserved in the China Veterinary Culture Collection Center (CVCC No. 102059). The spores were isolated from the silk glands of severely infected fifth instar silkworm larvae which were challenged at the third instar stage by feeding on mulberry leaves artificially contaminated with *N. bombycis* (approximately 2.0×10^5^ spores per larvae) [20]. The spores were purified with a discontinuous sucrose gradient (10, 25, 50, 75 and 90%, v/v) under aseptic conditions. The pellets of mature spores were rinsed twice with sterilized double distilled water and stored with antibiotics (100 µg/ml streptomycin, 100 U/ml penicillin ) for later use [18], [21]. In order to rule out bacterial contamination, purified spores were added to the normal cultured BmE cells. If no bacterial contamination was found at 48 hours post infection, the purified spores were suitable for the oral infection.

To clearly observe the purified *N. bombycis* spores, scanning electron microscopy was employed. Purified spores were first fixed with 2.5% glutaraldehyde in 0.1 mol/l PBS buffer (pH 7.4). Fixed spores were washed 3 times in PBS buffer at room temperature and then postfixed using 1.0% osmic acid, rinsed with PBS, subjected to a graded ethanol series (30, 60, 70, 80, 90, and 100%) and a graded tert-butyl alcohol series (50, 75, and 100%). Tert-butyl alcohol/acetonitrile (2∶1, v/v), tert-butyl alcohol/acetonitrile (1∶1, v/v) and acetonitrile were used to dry the spores separately. Finally, the spores were examined and photographed with a Hitachi S-3000N scanning electron microscope.

### Oral Infection of Silkworms by *N. bombycis* Spores

For insects, most pathogens invade hosts through food. Naturally, *N. bombycis* spores always enter into the midgut of silkworms with contaminated mulberry leaves, germinate under the alkaline environment and then lead to a destructive chronic disease. To develop a method close to the natural infection process of *N. bombycis*, the oral infection method previously applied was modified [19]. Briefly, approximately 120 third instar molted silkworm larvae were placed in a petri dish without food to maintain hunger before infection. *N. bombycis* spores were washed for three times with distilled water and then suspended at 10^7^ spores per ml. Approximately 600 µl of the spore solution and 6 grams of artificial diet were thoroughly mixed in a plate. The mixture was then cut into fine grains and given to the hungry silkworms. Four duplicates were simultaneously performed. The larvae were raised at 25°C with approximately 70% humidity and for approximately 5 hours before the larvae consumed the spore. To maintain a long period for spore persistence in the midgut, larvae were reared with normal artificial diet after 8 hours. Larvae of one group were collected at different time points after infection to perform microarray assay and quantitative real-time polymerase chain reaction analyses. The other three groups were counted at different time intervals to calculate the survival rate. The controls, uninfected larvae, were fed with the same amount of artificial diet and the same volume of sterile ddH_2_O under identical rearing conditions.

### Sample Preparation and RNA Extraction

Samples of infected larvae (i.e., the treatment set) and uninfected larvae (i.e., the control set) were collected at 2, 4, 6 and 8 dpi. Three larvae were collected as one sample and snap-frozen in liquid nitrogen. Three duplicate samples were obtained at each time point and stored at −80°C for RNA extraction. Total RNA was isolated using TRIzol reagent (Invitrogen, USA) and purified with NucleoSpin® RNA clean-up kit (MACHEREY-NAGEL, Germany). Total RNA templates were quantified by measurement of the 260/280 and 260/230 nm absorbance ratios with an ND-1000 spectrophotometer (NanoDrop Technologies, Rockland, DE, USA) and 1.0% formaldehyde-denatured agarose gel electrophoresis. The available samples were confirmed as having good integrity, and absorbance ratios of A_260/280_ were between 1.8 and 2.1. The samples were stored at −80°C for further analysis. The midgut samples of each set were also randomly dissected from three larvae for microscopy and RT-PCR analysis to verify the infection at each time points.

### RNA Labeling and Hybridization

RNA labeling and microarray hybridization was conducted by the CapitalBio Corp. (Beijing, China), and gene expression analysis was based on the Affymetrix Silkworm GeneChip kit in accordance with Affymetrix GeneChip expression operation procedures, which are found on the website of CapitalBio (http://www.capitalbio.com). After hybridization to the 23 K silkworm genome oligonucleotide chip (CapitalBio), containing 22,987 oligonucleotide 70-mer probes, the slides were rinsed with washing solution (0.2% SDS, 2×SSC) at 42°C for 5 min and washed with washing solution (2×SSC) at room temperature for 5 min [19]. The signals were scanned with a LuxScan 10 KA scanner (CapitalBio corp). Three biological replicates were conducted for each time point.

### Microarray Data Analysis

The images obtained by a LuxScan 10KA scanner were analyzed with LuxScan 3.0 image analysis software (CapitalBio). The global mean of each chip was first adjusted according to the global mean of the overall cy5 and cy3 signals across replicates, and then the signal intensity of each chip was normalized using the Lowess method [22]. The standard for gene expression was set at a signal intensity of more than 400. Significant analysis of microarray (SAM) was employed to select differentially expressed genes with an FDR <5% [23]. Only when a relative gene expression fold level was ≥2 and the P was <0.05 were genes considered to be up- or down-regulated in compared with control larvae.

The microarray data obtained in this study has been deposited to Gene Expression Omnibus (Accession number: GSE51247). The induced gene ontology analysis was performed using the Blast2go software and the online molecule annotation system from CapitalBio Corp. (MAS, 3.0, http://www.capitalbio.com). Online pathway relationship database KEGG (http://www.genome.jp/kegg/) was used to predict typical enzyme-catalysis reactions. Cluster analysis of induced genes was performed with the Gene Cluster 3.0 software by average linkage of hierarchical cluster analysis.

The silkworm gene sequences can be retrieved from the Silkworm Genome Database (SilkDB, http://silkworm.swu.edu.cn/silkdb/). The sequence of *Nbserpins* can be downloaded from Silkworm Pathogen Database (SilkPathDB, http://silkpathdb.swu.edu.cn/silkpathdb/). The microarray data and probe sequences were obtained from the *Bombyx mori* Microarray Database (BmMDB, http://www.silkdb.org/microarray/). Genes were considered to be expressed only when the value of the expression data was more than 400. Genes showing expression in a single tissue or more than 10 times than that of other tissues were considered tissue specific genes [19].

### Real-time Quantitative PCR

Total RNA samples from uninfected and *N. bombycis*-infected silkworm larvae at 2, 4, 6 and 8 dpi were the same as the total RNA used in microarray analysis. Quantitative real-time PCR amplification was performed in a 25 µL reaction volume using a SYBR Premix Ex Taq™ kit (TaKaRa) and 0.2 µM gene specific primers according to the manufacturer’s instructions. The results were captured with the ABI step one software (Applied Biosystems). Amplification was performed for three biological replicates at each time point, and sw22934 (transcription initiation factor 2 gene) was used as an internal standard [24]. The specific primers of genes randomly selected from all of the altered genes are listed in Table S1, and cDNA samples from the four time points for the induced and uninfected larvae were used for each pair of primers.

### Plasma Absorbance Detection

The third molted silkworm larvae were oral-infected by *N. bombycis*. Hemolymph samples were collected from infected silkworm larvae at 10 dpi and hemocytes were removed by centrifugation at 1,000 *g* for 5 min under 4°C. The plasma samples of the controls, uninfected larvae, were also obtained. The absorbance at 492 nm of the plasma was measured to quantify the levels of melanization at different time points and phenylthiourea-treated plasma was used as a negative control of the test. Three independent tests were performed.

## Results and Discussion

### Purified *N. bombycis* Spores Efficiently Cause Severe Pébrine in Silkworms

**Figure 1:**
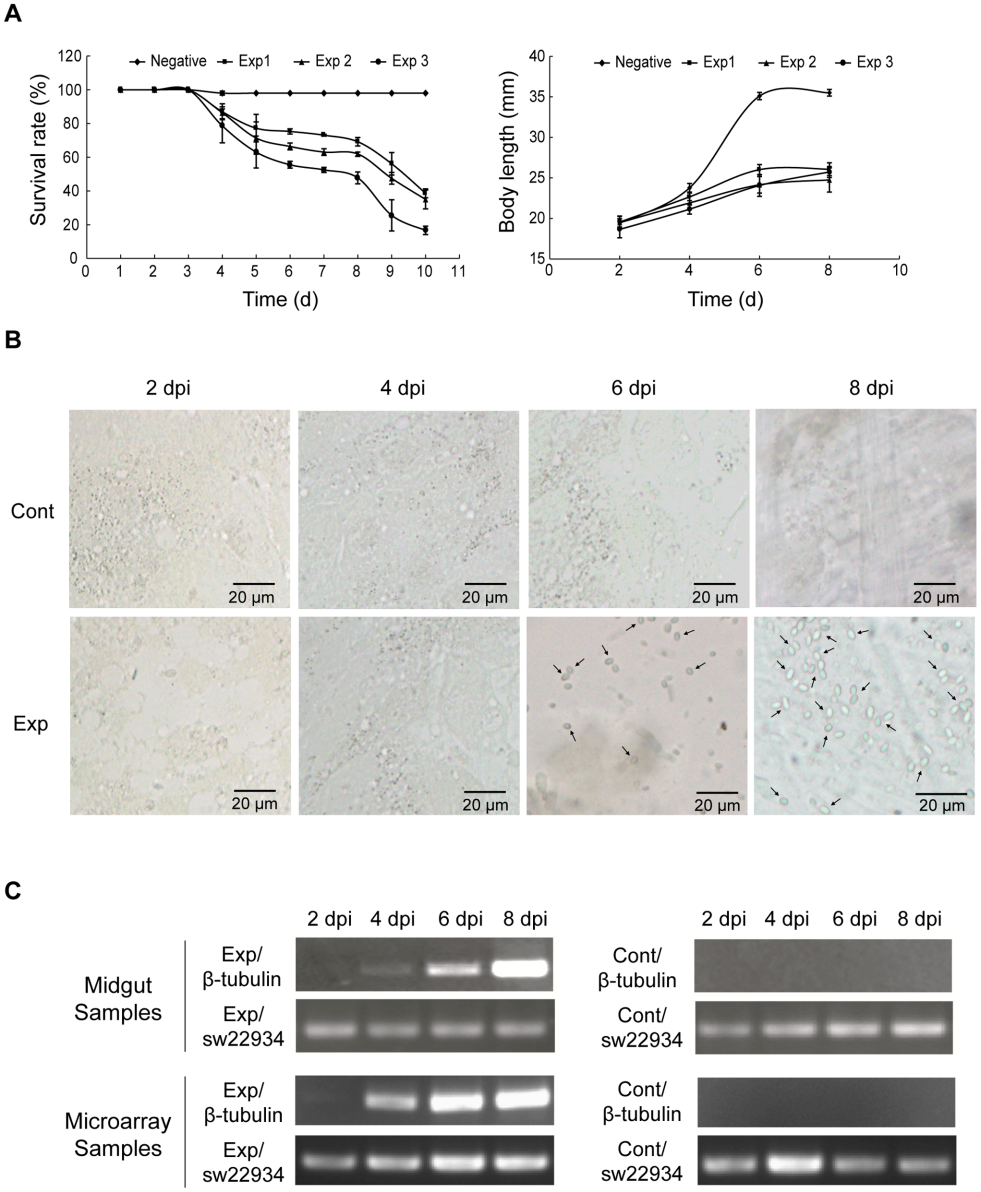
Observation and verification of *N. bombycis* oral-infected silkworms. (A) Survival curves and body length profiles of *N. bombycis* oral-infected silkworms. Negative: Uninfected silkworms reared under 25°C at 70% humidity. Exp 1,Exp2 and Exp3: silkworms fed with 5×10^4^, 1×10^5^ and 3×10^5^ spores per larvae, respectively. (B) Observation of spores in the midgut at different stages using optical microscopy. Cont: The midgut of an uninfected silkworm. Exp: Midgut from an infected silkworm. dpi: days post-infection. (C) Validation of infection by RT-PCR analysis. Midgut samples were randomly selected from the dissected larvae of *N.bombycis* infected set or the control set. The total RNAs for the analysis of microarray were also used to generate the sscDNA for RT-PCR to verify the infection. Exp: Infected larvae. Cont: Silkworm without infection. β-tubulin: A constitutively expressed protein coding gene (*β-tubulin*) of *N. bombycis*, Genbank ID: DQ663475.1. sw22934: A conserved silkworm housekeeping gene (*BmTIF*2), Genbank ID: NM_001043911.

**Figure 2:**
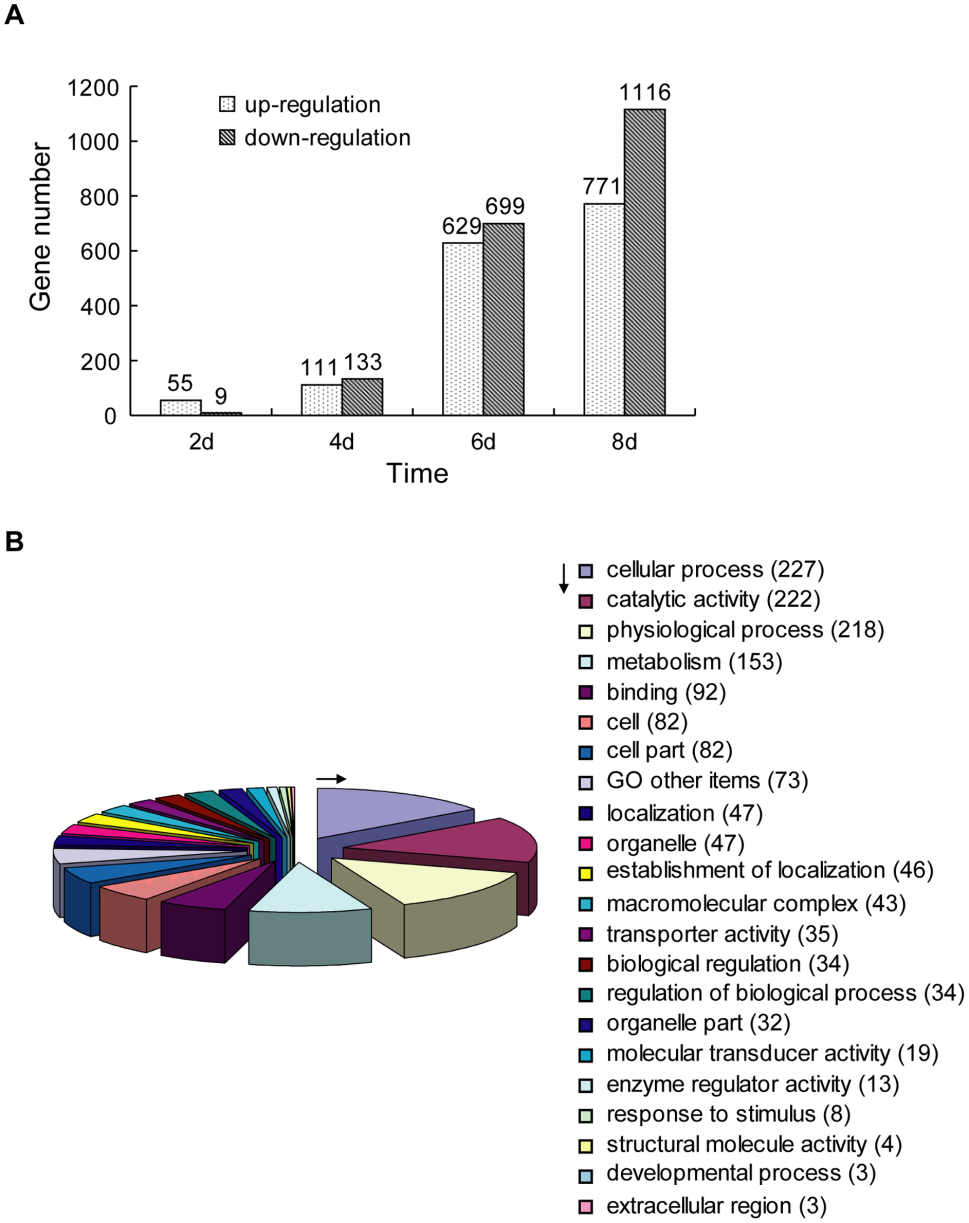
General statistics of the genes regulated after *N. bombycis* oral infection. (A) The number of up- and down-regulated genes after *N. bombycis* oral infection at the 2, 4, 6 and 8 dpi (d: days post-infection). (B) GO categories for all of the *N. bombycis* oral infection induced genes. The count of genes in each class was listed next to their name.

*N. bombycis* spores were first isolated and purified from infected silkworms (Fig. S1A). We determined the pathogenic ability of the purified spores using survival rate statistics for third-instar molted silkworm larvae after oral infection with CQ1 spores. After being fed with a discontinuous density gradient including 5×10^4^, 1×10^5^, 3×10^5^ spores per larva, pébrine occurred in all infected silkworms. The results showed a significant decrease in survival rate from 4 to 8 dpi. Furthermore, *N. bombycis* had a more than 50% mortality at 8, 9, 10 dpi upon infection (Fig. 1A). The remaining larvae died within 12 dpi under conventional rearing conditions. Obvious changes in the developmental physiology of the infected larvae observed were the following: 1) the body size of infected silkworms was drastically shorter than that of control larvae from 4 dpi (Fig. 1A); 2) the silkworm was noticeably smaller than the control set (Fig. S2). When examining the *N. bombycis* spore number under a microscope, *N. bombycis* could be observed in nearly all tissues of infected larvae, further confirming that the obtained *N. bombycis* spores had a strong pathogenic affect on its host (Fig. S3). Similar to spore infection under natural conditions, oral infection also results in typical disease symptoms such as slower development, prickly ash spots, molting difficultly, and dehydration (Fig. S1B). Based on the above observations, we concluded the strong, infectious and pathogenic ability of isolated spores is suitable for the further study.

### *N. bombycis* Induced a Strong and Complicated Host Response in Silkworms

After silkworm larvae were fed 5×10^4^ spores per larva, samples from infected larvae (i.e., the treatment set) and uninfected larvae (i.e., the control set) were collected at 2, 4, 6, and 8 dpi. Spore proliferation was detected by microscopic observation of the mid-gut (Fig. 1B). Meanwhile, RT-PCR was performed to determine the level of *N. bombycis*-specific constitutively expressed *β-tubulin* gene in the infected mid-gut (Fig. 1C) and result showed that *β-tubulin* expression was detected during the infection process. The above results indicated that all randomly selected larvae were infected, suggesting a widespread infection by *N. bombycis*. The infected silkworm exhibited a high mortality of up to 60% and had a distinct difference in body size compared with larvae in the control set.

Total RNA was extracted from the collected samples, and RT-PCR was performed to amplify *N. bombycis* specific gene (β-tubulin) to reconfirm the infection. It showed that *β-tubulin* was detected with a low expression level at 2 dpi (Fig. 1C), which could be attributed to the small amount of microsporidia exsiting in the infected larvae. Starting from 4 dpi, β-tubulin was significantly seen in the total RNA samples. The results suggested that the infection experiment was successful and the RNA samples were suitable for the further study. Then, RNA labeling and hybridization were processed by CapitalBio corp., and the 23 K silkworm genome oligonucleotide chip (CapitalBio) was employed. Using 2.0-fold as a cut-off, the transcription level of 2,691 genes (ca. 18.8% of total genes) was significantly altered between 2 and 8 dpi (Fig. 2A, Table S2). At the early stage of infection (2 dpi), there were 64 genes showing significant differences in terms of expression level based on our 2-fold cut-off criterion. Among these genes, 55 were up-regulated, whereas only 9 genes were down-regulated, suggesting that silkworm has a weak transcriptional response to *N. bombycis* at this stage. At 4 dpi, the expression level of more genes was altered suggesting that the host gradually responds to the invasion of *N. bombycis*. At 4 dpi, the number of down-regulated and up-regulated genes increased to 133 and 111 genes, respectively. At 6 dpi, a large number of genes were induced, indicating a stronger response to microsporidia invasion because there were 626 genes up-regulated and 699 genes down-regulated. This result is consistent with the view that *N. bombycis* has a growth cycle of approximately 4 days in insect cell lines. At the late stage of infection, microsporidia could be observed in most tissues of the silkworm, which can seriously disturb the metabolism and the growth and development of the larvae. Thus, the number of induced genes was higher than that found in earlier stages of infection. In total, there were 771 genes demonstrating up-regulation and 1,116 genes demonstrating down-regulation. These down-regulated genes include many immune- and metabolic system-related genes, indicating that infection has a strong inhibitory effect on the expression of host genes related to the immune response and basic metabolism.

Based on GO analysis of the differentially expressed genes, their functions could be classified into 22 categories (Fig. 2B). These data suggested that *N. bombycis* infection has an influence on a wide range of gene functions in the silkworm. Among these findings, genes involved in cellular process, catalytic activity and physiological processes were over-represented with differential expression found for 227, 222 and 218 genes, respectively.

**Figure 3:**
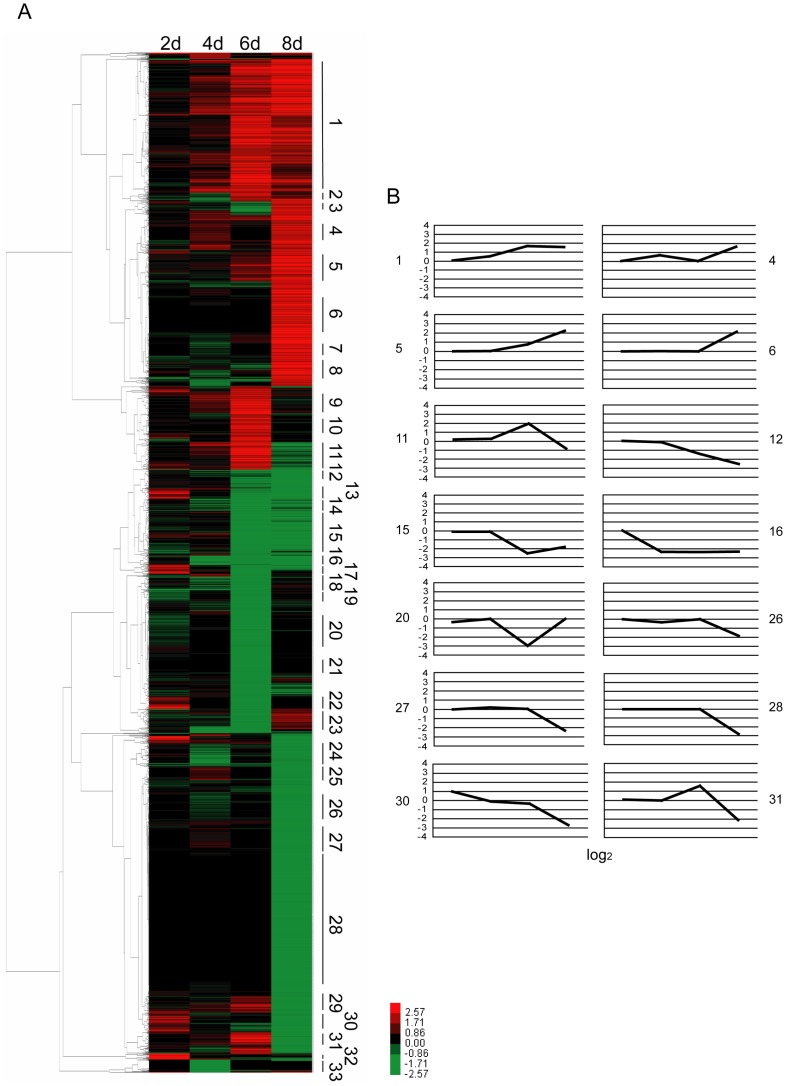
Differentially induced genes during *N. bombycis* infection. (A) Clustered expression profiles of the 2691 regulated genes with a 2.0-fold cutoff from 2 to 8 dpi. Expanded ratios and annotation details were list in Table S2. (B) Mean expression values for the induced genes (log_2_) located in the defined clusters. Genes involved in the 14 major clusters of induced expression profile analysis and the corresponding log_2_ value of their fold change were listed in Table S3.

In addition, cluster analysis of the induced genes was performed using a method previously described by LL Huang *et al* [19]. The average linkage hierarchical cluster analysis method in software Gene Cluster 3.0 was employed to demonstrated time-specific patterns. At least 33 clusters were recognized using the *N. bombycis* infection microarray data and approximately 14 main clusters of gene expression patterns were further analyzed (Fig. 3, Table S3). Among these clusters, clusters 1 and 16 were notable for their constant up- and down-regulation during the infection process. Clusters 6 and 28 were notable for their induction during the late stage of the invasion. Clusters 30 exhibited a dynamic exchange from up- to down-regulation over the course of the infection. Clusters 20 and 31 showed significant down- or up-regulation during the mid-stage of the infection. Cluster 5 and 12 were shown a significant up- or down-regulation starting from 6 dpi. All of these results exhibited various regulation profiles for the induced genes of silkworm larvae after *N. bombycis* invasion, indicating that a large number of genes are sensitive to infection by *N. bombycis*.

### *N. bombycis* Disturbed the Synthesis and Metabolism of Silkworm Juvenile Hormone

As a growth hormone that is synthesized by corpora allata, juvenile hormone (JH) is a sesquiterpene member that regulates the physiological and development processes of insect ecdysis, metamorphosis, reproduction and diapauses [25], [26]. The biosynthesis pathway for silkworm JH has been shown to consist of three different stages: derivation of isopentenyl diphosphate (IPP) from mevalonate pathway, conversion of IPP into farnesyl diphosphate (FPP), and finally, transformation of FPP into different types of JHs after various modifications [27]. In addition, JH can bind with juvenile hormone binding protein (JHBP) to regulate the expression of target genes or become metabolized into JH acid diol (JHad) or JH diol phosphate (JHdp).

**Figure 4:**
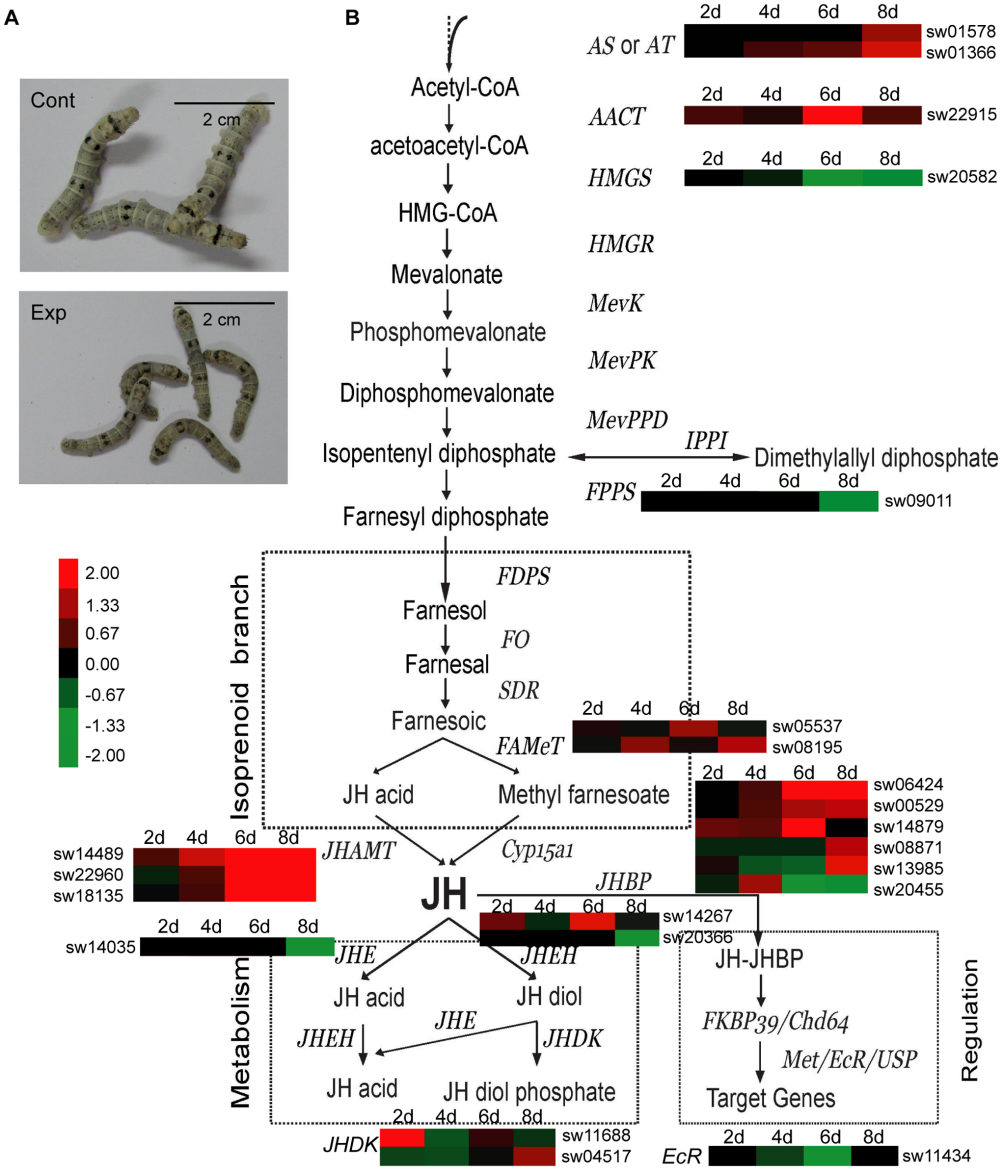
Diagram showing the induced biosynthesis, metabolism and signal transduction related genes of juvenile hormone in the silkworm. (A) Observation of silkworm infected by *N. bombycis* at 8 dpi. Cont: Uninfected silkworm larvae. Exp: Silkworm oral infected by *N. bombycis* spores. (B)The expression pattern for each gene is indicated near the gene name and the probe ID of BmMDB is shown aside. Diagram of the pathway is referenced from Daojun Cheng, 2008. See Table S4 for a detailed view of the cluster ratios.

In our analysis, the expressional level of many genes related to silkworm JH synthesis and metabolism were significantly altered (Fig. 4B, Table S4). The expression level of two allatostatin preprohormone (AS) genes (sw01578 and sw01366) that function in neuropeptide regulation, were up-regulated 2.3- and 3.1-fold, respectively, at 8 dpi. The acetoacetyl CoA thiolase (AACT) gene (sw22915), which encodes an enzyme that catalyzes Acetyl-CoA into IPP, was upregulated by at least 4.3-fold at 6 dpi. Two farnesoic acid O-methyltransferase (FAMet) genes, sw05537 and sw08195, which convert farnesoic acid to methyl farnesoic, were up-regulated more than 2-fold. JH acid methyltransferase (JHAMT) converts JH acids or JH precursors to active JHs in the last step of the JH synthesis pathway [28]. Our results showed that three JHAMT genes (sw14489, sw22960 and sw18135) were strongly up-regulated from 2 to 8 dpi, suggesting the accelerated synthesis of JH through the JH acid branch pathway after *N. bombycis* infection [29].

In addition, the process of JH metabolism was also affected by *N. bombycis* infection. The JH esterase (JHE) gene (sw14035), which encodes the enzyme that catalyzes JH to JH acid, was down-regulated from 2 to 8 dpi. In a previous study, the decrease in JH-esterase activity was reported as a possible explanation for juvenilizing the effect of *Nosema* implantation [30]. JH epoxide hydrolase (JHEH) has been shown to play a role in the catalysis of JH into JH diol [31]. One of the two JHEH genes was down-regulated at 8 dpi, whereas the other JEHE gene displayed a dynamic regulated status because its transcribed level showed up-regulation at 2 and 6 dpi and down-regulation at 4 and 8 dpi from 2 to 8 dpi. Interestingly, most of the JH binding protein (JHBP) genes (sw08871, sw06424, sw00529, sw14879 and sw13985) were up-regulated with the exception of one JHBP gene (sw20455), which was consistently down-regulated from 2 dpi to 8 dpi. The ecdysone receptor gene (EcR, sw11434), which is involved in JH signal transduction, was down-regulated during the infection.

Hormones are essential substrates in organisms that play important roles in the regulation of metabolism, growth, development, and reproduction. Here, *N. bombycis*-infected larvae had a slower growth rate than control silkworms (Fig. 4A). In fact, juvenilizing effect of microsoporidian infection has been known for many years [32]. The genus *Nosema* was reported to result in abnormal metamorphosis of saturniid moths, which were concluded for the excess juvenile hormone titre [33]. In this study, JH biosynthesis-related genes, including AS, AACT, FAMet and JHAMT, were up-regulated, but the expression of JH metabolism-related genes were mainly down-regulated after *N. bombycis* infection. We suspected that it disturbed the hormonal balance and may cause the increase of JH level in silkworm that acquired pébrine. Interestingly, a lot of JH-related genes could also be up-regulated after Nuclear Polyhedrosis Virus (NPV) and *Bb* oral infection [19]. In addition, synthetic JH can improve the production of NPV in insect cells [34]. Hence, we concluded that *N. bombycis* might disturb the silkworm JH synthesis and metabolism to retard the larvae development and provide time and nutrients for its reproduction, similar to NPV and *Bb*.

In higher vertebrates, hormones, nervous system and innate immunity collaborate through receptors on cells that enhance the immune-response during infection [35], [36]. Recently, JH and JH homologs were reported to counteract the immune response induced by 20-hydroxyecdysone (20-E ) in a *Drosophila* stable cell line [37]. Interestingly, 20-E has an antagonistic effect, but JH can stimulate the production of AMPs in the silkworm fat body [38]. Here, many of JH biosynthesis-related genes were up-regulated after *N. bombycis* infection, and we predicted that JH might be involved in the regulation of silkworm AMPs and function as a strategy for host resistance to microsporidia.

### *N. bombycis* Perturbed the Basal Metabolic Pathways of the Silkworm

Metabolism is the result of a plethora of chemical reactions that take place within each cell of a living organism that maintains normal physiological activities. We searched the KEGG database to select genes involved in host metabolic pathways using standard criteria for pathway prediction with a P<0.05 and induced ratios >2 or <0.5 [39]. We found that 124 basal metabolism-related genes, approximately 15.5% of genes associated with silkworm basal metabolism, were modulated after *N. bombycis* infection. Further, we found that eight genes involved in basic transcription were modulated, and seven were up-regulated from 2 to 8 dpi, indicating that the synthesis of nucleotides and proteins in silkworms is enhanced after infection (Table S5). Similarly, several silkworm basic transcription factors were up-regulated by *Bb* infection, including TFIID11, TFIID1, TFIIA1, TFIIE2 and TFIID10. After Madin-Darby bovine kidney cells infected by bovine viral diarrhea virus (BVDV), genes encoding proteins involved in protein translation and post-translational modifications were also found to be generally unregulated [19], [40]. The transcription genes upregulation after *N. bombycis* infection are consistent with those in silkworms infected with *Bb* and BVDV-infected Madin-Darby bovine kidney cells. Thus, providing further support for the theory of a general response after pathogen infection. Eight types of basal metabolism: nucleotide metabolism, amino acid metabolism, carbohydrate metabolism, genetic information transcription, xenobiotic biotransformation, cofactor and vitamin metabolism, lipid metabolism and glucoprotein and saccharometabolism were affected after infection. The detailed information and induced ratios for the involved genes are listed in Table S5. Most genes related to pyrimidine metabolism, amino acid metabolism, genetic information transcription, xenobiotic biotransformation, and cofactor and vitamin metabolism were significantly up-regulated during the infection process (Table S6). *N. bombycis* consistently induced sublethal effects on the host together with a chronic infection. Acceleration of these basal metabolic processes may be due to the demands of *N. bombycis* reproduction. A similar up-regulation was also found during a study on silkworms challenged with *Bb* [19].

### *N. bombycis* Induced a Wide Silkworm Immune Response

In contrast with vertebrates, insects lack a specific adaptive immune system and have a simple, efficient nonspecific innate immune system for resisting the invasion of pathogens [41]. Humoral and cellular immunity serve as the main parts of insect immune systems and play an important role in pathogenic microorganism clearance [42]. It is worth noting that melanin formation in the hemolymph after pathogen infection is universal and melanization is an important part of insect immune defense system.

### Silkworm Humoral Immunity

**Figure 5:**
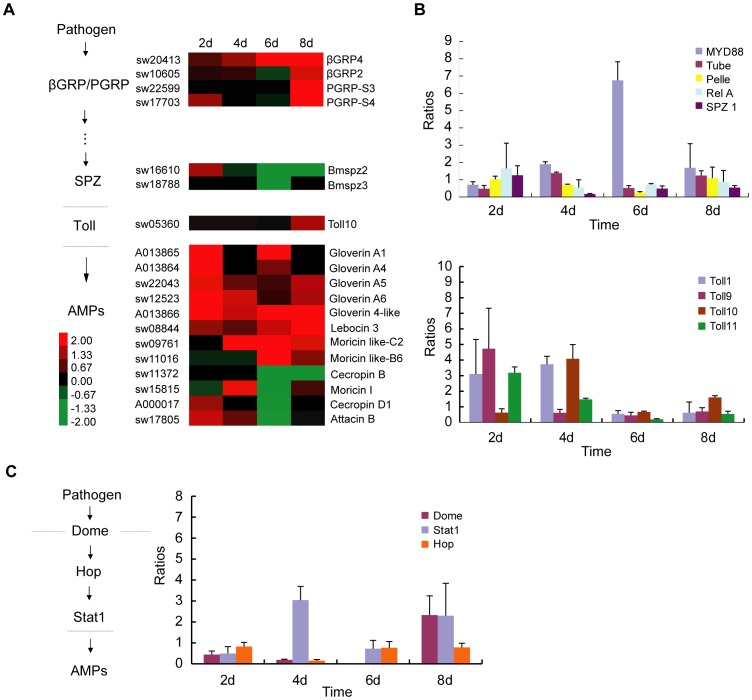
*N. bombycis* induced a silkworm systemic immune response. (A) A diagram of the Toll signal transduction pathway and *N. bombycs* induced expression of the related genes. The probe ID of BmMDB or gene ID of SilkDB are shown on the left (for each gene ID, we used the letter A to substitute letters BGIBMGA to simplify the expression). (B) qRT-PCR of genes related to the silkworm Toll pathway. (C) Sketch of the JAK/STAT signal transduction pathway and qRT-PCR analysis of the corresponding genes. See Table S4 for a detailed view of the cluster ratios.

The main characteristics of insect humoral immune response include secretion of antimicrobial peptides for defense against the invasion of pathogenic microorganisms [43]. In *Drosophila melanogaster*, Toll/dif pathway and Imd/relish are the main components of its innate immune system and play an important role in the regulation of the expression of antimicrobial peptides [44]. For example, fungi and gram-positive bacteria can induce the activation of the Toll/dif pathway and regulate the expression of drosomycin, metchnikowin and defensin in fruit fly. Meanwhile, gram-negative bacteria can activate the Imd/relish pathway and lead to an increase in the expression of cecropins, drosocin, diptericins and attacin [45], [46]. The completion of the genome sequence and microarray-based gene expression profile of *Bombyx mori* allowed us to diagnose the host immune response to *N. bombycis* infection [17], [18]. Similar to *Drosophila*, the silkworm humoral immune system is also mainly composed of Toll, Imd and JAK/STAT pathways, which could be induced by pathogenic microorganisms [47], [48], [49], [50]. *N. bombycis* can invade silkworm *B. mori* and develop a sublethal infection despoiling nutrient substances from its host and causing cytopathic necrosis [1]. To our knowledge, the molecular mechanism for the host response to *N. bombycis* infection remains elusive. To better understand the effects of *N. bombycis* infection on its host immune system, we analyzed the microarray data for the silkworm genes involved in the Toll, Imd and JAK/STAT signaling pathways (Table S4). The expression of β-glucan recognition protein 4 (β-GRP4) was up-regulated throughout the infection process (Fig. 5A), while β-GRP2, PGRP-S3 and PGRP-S4 were significantly up-regulated at the late stage of infection (8 dpi), suggesting an important role in pathogen recognition after a severe infection. β-GRP4 (sw20413), which is highly expressed in the midgut, separately represented approximately a two- and four-fold increase in up-regulation at 4 and 6 dpi, and its expression reached the highest level at 8 dpi, which is approximately a five-fold increase in up-regulation. This result indicated that β-GRP4 played a role in recognizing initial invasive spores and transmits a signal to the downstream cascade reaction in the midgut. β-GRP2 (sw10605), which is expressed at high levels in the midgut, integument, hemocyte and fat body, showed an almost three-fold up-regulation. Two peptidoglycan recognition short-type proteins, PGRP-S3 (sw22599) and PGRP-S4 (sw17703), were strongly induced after the *N. bombycis* infection at 8 dpi. PGRP-S3 and PGRP-S4 are only expressed in the midgut, and both showed an approximate eighteen-fold increase in up-regulation, indicating that *N. bombycis* spores are recognized by PGRP in silkworm midgut at the late stage of infection. However, no long-type PGRPs were modulated in this study.

When pathogens are recognized by insect immune system, signals are delivered to downstream signaling pathways and lead to the release of antimicrobial peptides. Mature spatzle, which are proteolytically processed after activation by activated recognition factors, binds as a dimer to Toll receptors to transmit signals into the cytoplasm, which will ultimately lead to MyD88, Tube and Pelle recruitment [51]. Finally, the Rel transcription factors will be released to induce AMPs expression [19]. In this analysis, *SPZ2* (sw16610) was found to be up-regulated at the early stage of microsporidia infection and showed nearly a 2.4-fold increase (2 dpi). *Toll 10* (sw05360) was the only Toll receptor that was found to be up-regulated at 8 dpi. Using the current selection standard, microarray data showed no significant modulation of other Toll pathway-related genes such as *MyD88*, *Tube* and *Rel*, because the signal value for most of the genes was less than 400. Thus, real-time PCR was employed to analyze the regulation of the genes involved in the silkworm Toll pathway (*SPZ1*, *Toll1*, *Toll9*, *Toll10*, *Toll11*, *Myd88*, *Tube* and *Pelle*) at 2, 4, 6 and 8 dpi (Fig. 5B, Table S7). The results showed that *Toll1, Toll9, Toll10, Toll11, Myd88, Tube* and *Pelle* are up-regulated after *N. bombycis* infection. *Toll* receptors *Toll 9* and *Toll 11* were highly induced at the beginning of the infection (2 dpi) approximately four-fold, while *Toll 1* and *Toll 10* showed marked up-regulation at 4 dpi, almost four-fold more than control. The adapters *MyD88*, *Tube* and *Pelle* were also increased, and *MyD88* peaked at 6 dpi with six-fold up-regulation. Surprisingly, *SPZ1* was not induced after *N. bombycis* infection, which is indicative of its absence from the immune response to the microsporidia in silkworm. All these results indicated that the silkworm Toll pathway was activated by *N. bombycis* infection.

JAK/STAT pathway was first indentified in *Drosophila* and verified to be involved in the immune response [52], [53]. Receptors Domeless and JAK Hopscotch and the STAT transcription factor are the major contributors in this pathway [54]. In addition, the pathway was demonstrated to be involved in antiviral and inflammatory responses [55], [56]. In our study, real-time PCR was conducted to examine the expression of genes involved in the silkworm JAK/STAT pathway (*Dome*, *Hop* and *STAT1*) at 2, 4, 6 and 8 dpi (Fig. 5C, Table S7). The results showed that Hop was not activated during the infection process. However, notably, *STAT1* was up-regulated at 4 and 8 dpi approximately 3-fold. *Dome* was also found to be up-regulated, approximately 4-fold, at 8 dpi. These data suggest that *N. bombycis* infection induces the JAK/STAT pathway in silkworm. In *Drosophila*, Imd pathway is activated by gram-negative bacteria infection [57], whereas no significant modulation related to this pathway was found in our microarray data.

After infection, twelve AMP genes belonging to the Gloverin, Cecropin, Moricin, Attacin and Lebocin families were induced, indicating activation of the silkworm systemic immune response. Five genes in the Gloverin subfamily (BGIBMGA013866, BGIBMGA013865, sw22043, sw12523, and BGIBMGA013864) showed a significant increase in gene expression level. The members of the Lebocin and Moricin subfamilies, including Lebocin 3 (sw08844), Moricin like-C2 (sw09761) and Moricin like-B6 (sw11016), were also up-regulated after *N. bombycis* infection. These results indicate that the *N. bombycis* spores were recognized by GNBPs and PGRPs and that the Toll signaling and JAK/STAT pathways were activated to release AMPs.

Insect humoral immune is one of the main insect immune responses that can efficiently resist the invasion of microorganisms. Here, we found an important factor SPZ2 but not SPZ1, involved in silkworm Toll pathway was up-regulated at the early stage of infection (2 dpi). It suggests a potential function of SPZ2 in the activation of downstream Toll receptors during *N. bombycis* infection. Meanwhile, real-time PCR analysis suggested that the Toll signaling and JAK/STAT pathways were activated to participate in *N. bombycis* infection. The AMP families, including Gloverin, Moricin and Lebocin, were strongly induced from 2 to 8 dpi; however, in *Apis mellifera*, the infection of *N. ceranae* led to significant host immunosuppression by down-regulation of defensin, abaecin, apidaecin and hymenoptaecin [58].

### Silkworm Phenoloxidase Cascade Melanization

**Figure 6:**
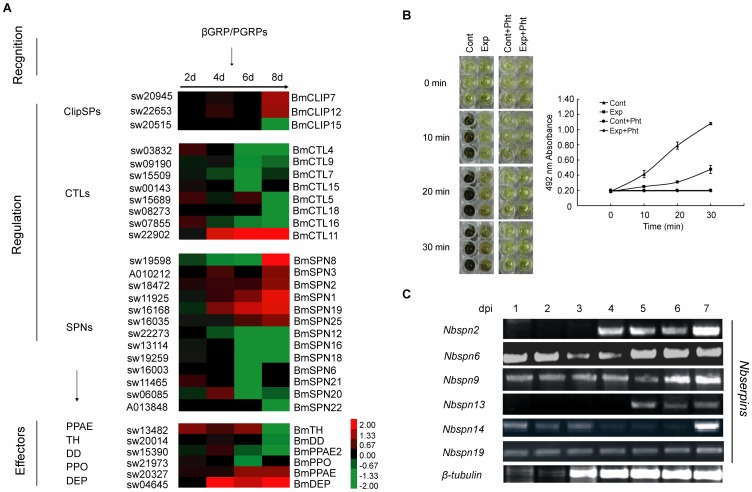
*N. bombycis* perturbed the silkworm serine protease cascade melanization pathway. (A) Cluster diagram of the induced genes involved in the serine protease cascade melanization pathway. See Table S4 for a detailed view of the cluster ratios. (B) Measurement of the melanization speed of silkworm plasma. The silkworms were oral-infected by *N. bombycis* and hemolymph was collected at 10 dpi. The cell-free plasma was obtained and three replicates were performed. The averages represented the three replicates from one experiment and the error bars in the graph indicated the standard deviation (SD) value. Cont: Uninfected silkworm larvae. Exp: Silkworm treated with *N. bombycis* spores. Pht: phenylthiourea. (C) Transcriptional analysis of of *N.b.serpins* using RT-PCR. *Nbserpin2* (SilkPathDB No. NBO_18g0004), *Nbserpin6* (NBO_34g0030), *Nbserpin9* (NBO_39i001), *Nbserpin13* (NBO_124g0001), *Nbserpin14* (NBO_372g0002), *Nbserpin19* (NBO_1570g0002).

Melanization played an important role in wound healing, encapsulation and microorganism- degradation [59], [60]. Phenoloxidase cascade melanization is an effective system including two main steps: first, recognition of the foreign target or the wound site as foreign. Second, one or more hemocytes are activated to perform a particular effector response [61]. A previous study on silkworms infected by *Bb* suggested that the serine protease cascade melanization pathway is significantly activated after infection [19]. Unexpectedly, most of the genes associated with the silkworm serine protease cascade melanization pathway were down-regulated in our data (Fig. 6A, Table S4). The increased expression of pattern recognition receptors, such as *βGRP2*, *βGRP4*, *PGRP-S3* and *PGRP-S4*, showed that silkworm had initially recognized the entered microsporidia and may have transmitted the signal to the insect innate immune system (Fig. 5A). After the recognition of pathogens, CLIP serine proteases (*CLIPs*) and serpins (*SPNs*), which serve as regulators of the serine protease cascade, were induced. Three *CLIP* genes were selected here, and two were up-regulated i.e., *CLIP7* (sw20945), *CLIP12* (sw22653), but *CLIP15* (sw20515), showed an opposite pattern. In addition, 13 of the 34 silkworm *SPN*s were altered, and 8 were down-regulated. Only the expression level of *SPN1* (sw11925), *SPN2* (sw18472), *SPN3* (BGIBMGA010212), SPN19 (sw16168) and *SPN25* (sw16035) was increased. CTLs were also involved in the regulation of insect melanization [62]. The 8 C-Type lectin encoding genes were modulated, and 7 of the 8 genes were down-regulated. Only the expression of *CTL11* (BGIBMGA006623), which is highly expressed in the silkworm fat body, integument and gonads, was strongly induced from 4 to 8 dpi and increased more than 17-fold at 8 dpi. In addition, several key enzymes during the silkworm melanization process, such as phenoloxidase-activating enzyme (BmPPAE, sw20327), BmPPAE2 (sw15390), dopa-decarboxylase (BmDD, sw20014) and tyrosine hydroxylase (BmTH, sw13482) showed modulation. Three of these enzymes with the exception of BmPPAE performed an up to down-regulation profile. BmPPAE, which was highly expressed in the head and hemolymph, and it was consistently up-regulated from 2 to 8 dpi. These findings indicate that BmPPAE is important in hemolymph melanization toward the invasive *N. bombycis*. PPO, served as a melanization effector that was induced, and PPO1 (sw21973) was down-regulated at 6 dpi. These results showed that the expression of many genes associated with the melanization pathway was suppressed after *N. bombycis* invasion, particularly from 6 to 8 dpi.

Insect melanization is an important and special congenital immune defense mechanism that is caused by the serine protease cascade melanization pathway after pathogen invasion. After the infection of *N. bombycis*, silkworm larvae often acquired the formation of dark, melanized spots or areas on the cuticle. But no similar situation was observed in other infected tissues, such as fat body, midgut and silkgland. In this data, genes of the serine protease cascade in melanization pathway were induced but finally the key effectors (BmDD, BmTH and BmPPO) involved in the pathway were down-regulated. A suppression of melanization by microsporidia occurred and it suggested the host encountered the loss of efficient melanization as a result of *N. bombycis* infection. A test towards the melanization speed of silkworm plasma was completed. The absorbance at 492 nm of the plasma was measured to quantify the levels of melanization, and the phenylthiourea-treated plasma was used as a negative control for the assay. Interestingly, the result showed that the plasma isolated from *N. bombycis* infected larvae darkened slower than the control (Fig. 6B), which is consistent with previous analysis. Serpins are a broadly distributed family of protease inhibitors that use a conformational change to inhibit target enzymes [63]. They are central in controlling many important proteolytic cascades, including the insect serine protease cascade in melanization pathways [60]. In *N. bombycis* genome data, 19 serpin genes were indentified and 6 of them were predicted as secreted serpins, which may be involved in regulation of the host serine protease cascade in melanization pathway [16]. Here, we surveyed the transcriptional level of 6 *N.b. serpins* at 1 to 7 dpi in the mid-gut of *N. bombycis* infected silkworm (Fig. 6C). Interestingly, *Nbspn6*, *Nbspn9*, *Nbspn14* and *Nbspn19* were transcribed persistently during the infected process. *Nbspn*2 and *Nbspn13* were highly expressed at the later stage of infection. The expression of *N.b. serpins* of *N. bombycis* may suppress the proteolysis of PPO to active PO and finally cause a suppression of melanin formation. It may become a reason why the plasma of infected larvae had slower darkening.

Meanwhile, defense protein (DEP, sw04645), which was isolated from silkworm fat body and contained a reeler domain, was verified to be involved in the nodulation response and enhance phenoloxidase activity [64], indicating an important role of DEP in the prophenoloxidase activation cascade. Here, the expression of DEP was significantly increased after the infection and up to ∼11 fold at 8 dpi. This suggested that the silkworm was trying to increase the PO activity to defend the microsporidia, however, the down-regulated expression of *BmTH*, *BmDD* and *BmPPO* caused the formation of melanin to remain low in level. Thus, in the infected group, we observed a few infected silkworms with typical dark spots on the larvae cuticle, like the pepper scattered. The main part of silkworms showed no pepper lesion on the cuticle. However, insect melanization is a complex process during the immune response, more evidence is needed to decipher the mechanism of silkworm to resist *N. bombycis* infection.

### Silkworm Cellular Immunity

**Figure 7:**
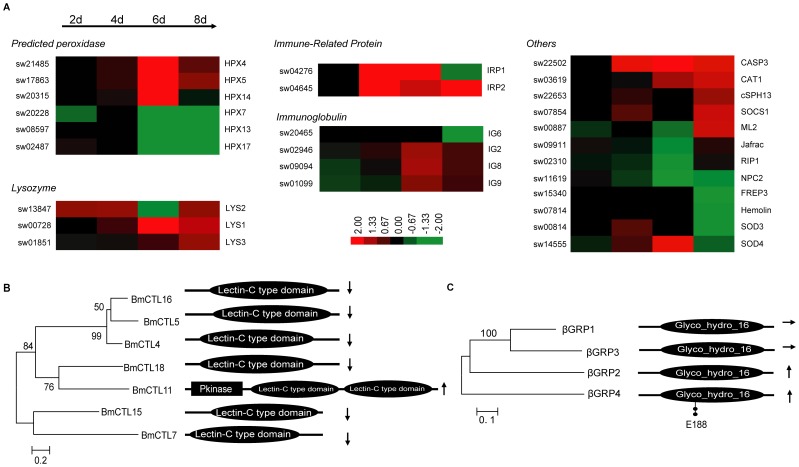
*N. bombycis* induced a silkworm cellular immune response. (A) Cluster of cellular immune response families. See Table S8 for a detailed view of the cluster ratios. (B,C) The phylogenetic analysis and domain prediction of *N. bombycis* induced BmCTLs and βGRPs. The up-regulated genes were indicated by the arrows pointing upward. The arrows pointing downward showed the down-regulated genes. If the genes were not induced in our data, horizontal arrows were used to represent them.

The innate immune system in insects is divided into humoral and cellular defense responses. Cellular defenses refer to hemocyte-mediated responses, such as phagocytosis and encapsulation. Many immune factors: lysozyme, scavenger receptors (SCRs) and lectins, are involved in the cellular immune response [65]. After the infection of *N. bombycis*, the cellular immune response is triggered (Fig. 7A). Three lysozyme (LYS) genes (sw00728, sw13847 and sw01851) were induced, all had approximately 2-fold up-regulation at 8 dpi. A similar phenomenon was seen in *Drosophila* flies infected by *Octosporea muscaedomesticae* [66]. Chintin is known as a major component of the spore wall of *N. bombycis* spores and many lysozymes have chitinase activity. It has been suggested that the induced lysozymes disturb the formation of the spore wall. Immunoglobulin superfamily (IgSF) proteins are known for their ability to specifically recognize and adhere to cells [67]. Four immunoglobulin encoding genes, *IG2* (sw02946), *IG6* (sw20465), *IG8* (sw09094) and *IG9* (sw01099), were induced, and three were up-regulated, suggesting a function for binding to the spores and neutralizing harmful effects [68]. Several members of cellular immune-associated gene families, such as the coding genes for immune-related proteins (IRPs), SOD, peroxidase (HPX) and caspase (CASP) were also induced (Table S8), indicating that an extensive cellular response occurred to cope with the infection of *N. bombycis*.

Lectins are sugar-binding proteins that play a role in biological recognition and cell adhesion. They can facilitate the condensation of infectious microorganisms during the immune response [44], [62], [69]. *CTL* genes are also involved in the regulation of insect melanization. In our data, eight of the 22 identified C-type lectin genes were modulated, and 7 were down-regulated. Only the expression of *CTL11* (sw22902) was persistent up-regulation during the *N. bombycis* infection suggesting that CTL11 performs a special promotion in immunoresponse to *N. bombycis* invasion. In order to explore the differences among these induced CTLs in sequences, a phylogenetic tree was reconstructed using MEGA 4.0 with 1,500-times bootstrap sampling. Domains were predicted by pfam software (Fig. 7B). Results showed that only CTL11 had a conserved protein kinase domain besides two lectin-C type domains. In *Arabidopsis thaliana*, a new class of putative plant receptor kinase with an extracellular lectin-like domain was discussed with regard to the transduction of oligosaccharide and plant hormone signals [70]. A receptor-like protein kinase with a lectin-like domain from *Populus nigra var. italica* was shown to be expressed in response to wounding and perform phosphorylation activity [71]. The up-regulation of *CTL11* suggests that it plays an important role in both spore recognition and immune signal transduction. Further studies with regard to CTL11 functionality will help us to understand the recognition and immunity of *N. bombycis.* Moreover, it has become a potential target gene to elevate the silkworm disease resistance.

Similarly, we performed phylogenetic analysis and domain prediction of βGRPs, HPXs and BmSPNs (Fig. 7C and Fig. S4). The results showed that β-GRPs all possessed glycosyl hydrolases family 16 domian. β-GRP4 was clustered into one clade and predicted to have the catalytic residues (E188). During the infection, β-GRP4 was persistently modulated from 2 to 8 dpi. The catalytic residues may play an important role in recognition of the microsporidia. A further study with regard to the interaction between β-GRP4 and *N. bombycis* will be crucial to further understanding of host recognition of invasive spores. However, the sequence analysis of HPXs and BmSPNs show no special characteristics among the induced members. Thus, a detailed and integrated analysis of the induced genes involved in immune-related gene families is necessary.

### Preliminary Identification of *N. bombycis* Specific-induced Immune Factors

**Figure 8:**
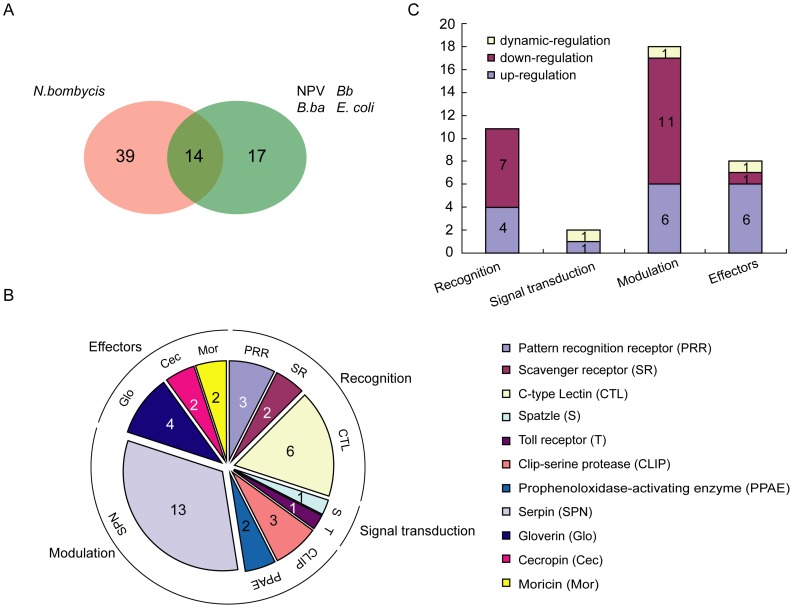
Analysis of *N. bombycis* specifically induced immune-related genes. (A) Number of induced genes common or unique in the different infections. NPV, *Bb* and *B.ba* represented nuclear polyhedrosis virus, *Bacillus bombyseptieus* and *Beauveria bassiana*, respectively. (B) Distribution of induced genes according to their functional categories in silkworm immune pathways. (C) The induced patterns of specific genes involved in different functions. See Table S9 for the ratio of silkworm immune-related genes induced by different pathogens.

A survey on silkworm gene responses to *Bb*, nuclear polyhedrosis virus (NPV), *Escherichia coli* (*E. coli*) and *Beauveria bassiana* (*B. ba*) was reported by LL Huang *et al,* who also used 23 K silkworm genome oligonucleotide chip (CapitalBio) [19], [72]. When we compared our results with the studies of LL Huang *et al*, we observed striking differences. After parasitic infection, 70 immune-related genes were recorded as significantly induced (listed in Table S9). Out of these, 14 immune genes were in common with the previous study, while 39 are *N. bombycis* specific induced genes (Fig. 8A). The *N. bombycis*-specific genes are found in different roles involved in recognition, signal transduction, modulation and effectors (Fig. 8B). These genes also had diverse expression patterns throughout the course of infection (Fig. 8C). Scavenger receptors are a group of receptors that recognize modified low-density lipoprotein and known as analogue to pattern recognition receptors of mammals. In insect, SCRs also work for pathogen recognition in the innate immune system [73]. One class B SCR gene, known to be involved in the phagocytosis of microorganisms in *Drosophila*, BmSCRB9 (BGIBMGA011408) was specifically upregulated after infection. BmSCRB9 was speculated to have a role in *N. bombycis* recognition during the infection. Moreover, a set of CLIPs and SPNs, which are important early regulators in insect immune responses, were also specifically induced. About 69% and 40% of specific SPNs and CLIPs were down-regulated, respectively. Interestingly, the gene expression of all the *N. bombycis* specific-induced *Gloverins* (BGIBMGA013866, BGIBMGA013865, BGIBMGA005658, and BGIBMGA013864) and *Moricins* (BGIBMGA011521, sw09761) were up-regulated. These results suggest that the AMP families of Gloverin and Moricin act as the major effectors to resist the infection by *N. bombycis*. Here, the preliminarily identified *N. bombycis*-specific response factors could serve as candidate genes for the selection of transgenic silkworm with high resistance to *N. bombycis* or as structural models for anti-microsporidia drug design. However, it was difficult at these results to draw firm conclusions, because different intervals of sample collection were used in these two studies, which could have given different responses.

### Real-time Quantitative PCR Analysis

**Figure 9:**
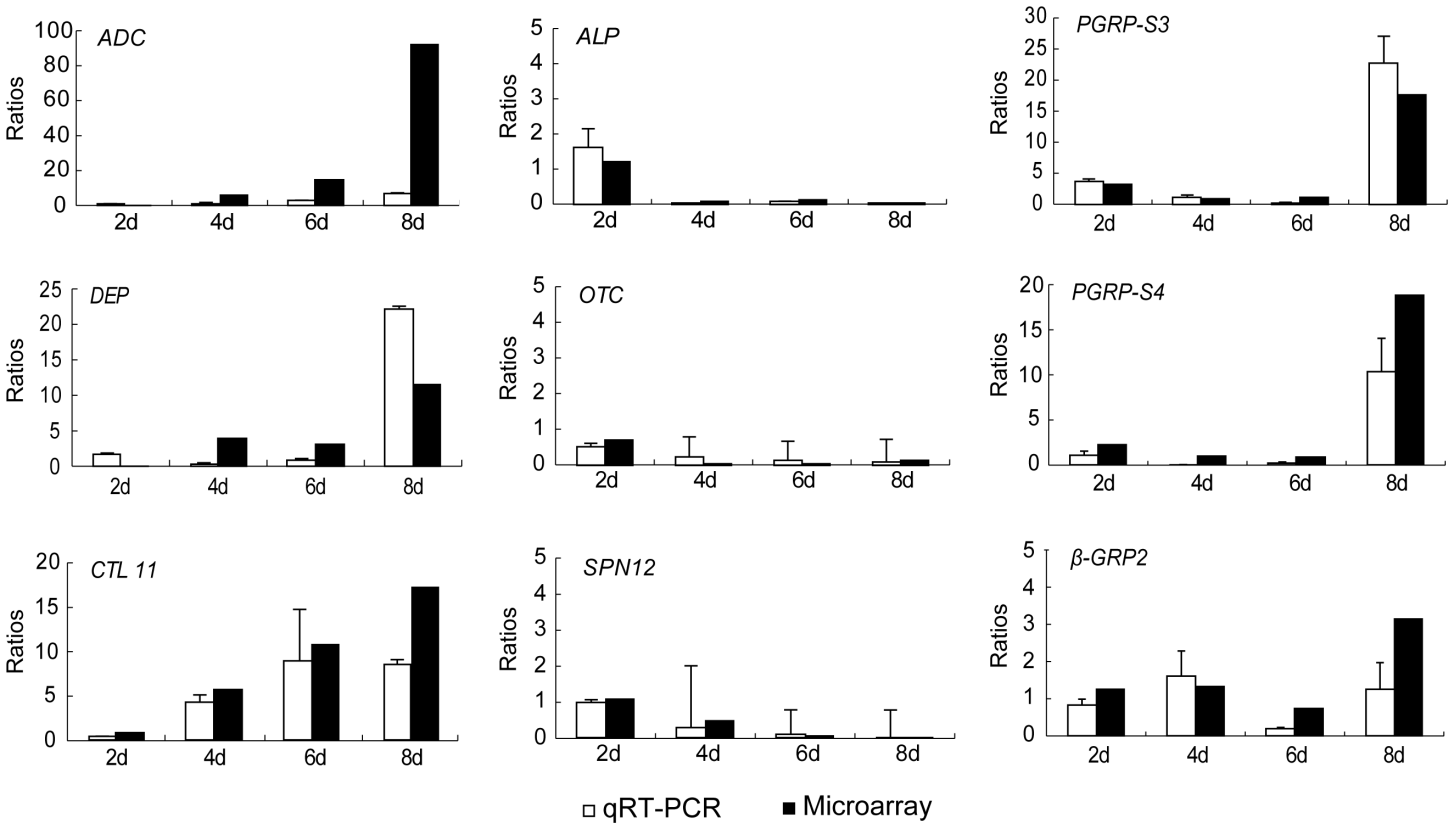
Real-time quantitative PCR was used to validate the differential expression of genes measured by microarray. A total of 9 genes, including peptidoglycan recognition protein-short 3 (*PGRP-S3*), *PGRP-S4*, β-glucan recognition protein 2 (*β-GRP2*), out domain containing protein (*OTC*), serpin12 (*SPN12*), C-type lectin 11 (*CTL11*), defense protein (*DEP*), adenylate cyclase (*ADC*) and alkaline phosphatase (*ALP*), at four time points was selected for primer design. The detailed sequences of the related primers are listed in Table S1.

To validate the differential expression of genes measured by microarray, 9 genes were randomly selected from the induced genes for real-time quantitative PCR analysis: peptidoglycan recognition protein-short 3 (PGRP-S3), PGRP-S4, β-glucan recognition protein 2 (β-GRP2), out domain containing protein (OTC), SPN12, CTL11, DEP, adenylate cyclase (ADC) and alkaline_phosphatase (ALP). Our results indicate that the tendency of the expression level of the 9 genes at four different time points had pattern similar to those measured by microarray analysis (Fig. 9), thus, confirming the accuracy of the results of microarray data in this study.

## Conclusions

**Figure 10:**
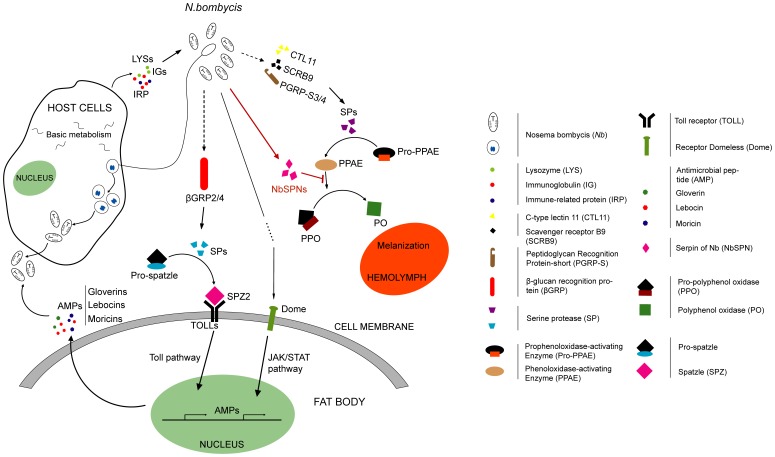
Schematic overview of the process for silkworm recognition and response to the invasion of *N. bombycis*. After *N. bombycis* oral infection, the invasive spores affected the expression of genes involved in the JH biosynthesis and metabolism pathway and may perturb the silkworm hormonal balance. In addition, the basal metabolisms of host cells were disturbed and spores execute the life cycle at intra-cellular. To combat proliferous microsporidia, the silkworm activated Toll and JAK/STAT signaling pathway and secreted the antimicrobial peptides. The serine protease cascade melanization pathway was also induced, but the secreted serpins of the invasive *N. bombycis* may participate in inhibiting the activity of silkworm serine proteases and then disturb the conversion of PPO to active PO. Meanwhile, the cells could also release the cellular immune effectors, such as lysozymes, IRPs and immunoglobulins to manage the microsporidia.

We challenged silkworm with *N. bombycis* isolate CQ1 and obtained the microarray data at 2, 4, 6 and 8 dpi. With subsequent analysis, we captured the molecular events defining the host response to *N. bombycis* infection (Fig. 10). A complex and strong host response in silkworm was induced. The invasion of *N. bombycis* altered the expression of genes involved in JH biosynthesis and metabolism pathway, which could affect the physiological and development process of ecdysis and the metamorphosis of infected silkworm larvae. In addition, eight types of basal metabolism were significantly modulated after the infection of *N. bombycis*. The enhancement of the basal transcription level meets the requirements for the host silkworm and microsporidia. To combat proliferous microsporidia, silkworm activated complex immune responses as follows: 1) β-GRP2/4 participated in recognition of invasive *N. bombycis* and triggered Toll signaling pathway. Meanwhile, membrane receptor *BmDome* was up-regulated and induced the response of JAK/STAT pathway. 2) The effectors of systemic immunity, particularly the AMPs (gloverins, lebocins and moricins) showed up-regulation during the infectious progress. 3) silkworm serine protease cascade melanization pathway was also induced, but a loss in effective melanization limited the pathogen clearance ability of the silkworm. The secreted serpins of the invasive *N. bombycis* may be involved in inhibiting the activity of silkworm serine proteases and then disturbed the formation of dopamine melanin. 4) Cellular immune effectors, such as lysozymes, immune-related proteins and immunoglobulins were also up-regulated to manage the microsporidia.

In summation we found an integral host response at the transcriptional level to microsporidia infection and preliminary identified the *N. bombycis* specifically induced immune-related genes. We found *N. bombycis* infection increased the rate of basal metabolism and disturbed the synthesis and metabolism of silkworm JH. Moreover, we obtained the evidences that Toll, JAK/STAT and melanization pathway were all induced during the *N. bombycis* infection, which is helpful to construct a global comprehension of molecular immunoresponse. Some important genes involved in pathogen recognition, immunoregulation, and immuoeffectors were obtained, and our results also suggested that the melanization pathway could be perturbed by the pathogen secreted proteins, providing interesting clues for further research on host-parasite interaction. The identification of *N. bombycis*-specific response factors could provide structural models for anti-microsporidia drug design or candidate genes for generating transgenic silkworms with high resistance to *N.bombycis*. Additionally, 90% agricultural pests belong to Lepidoptera, silkworm as the model of Lepidoptera; thus, allowing us to explore the potential drug target proteins in silkworm immunoresponse to intracellular parasites as the vulnerability of pests, which can also be utilized in pest control.

## Supporting Information

Figure S1 **Observation of purified spores and** ***N. bombycis*** **oral-infected silkworms.** (A) *N. bombycis* spores as visualized by optical and electron microscopes. (B) Oral infection with isolated *N. bombycis* causes severe pébrine, which is similar to natural infection. Cont:Uninfected silkworm larvae. *N. bombycis* infected silkworm larvae exhibited prickly ash spots on the cuticles and *N. bombyci*s-infected silkworms molted with difficultly. The disease symptoms were similar to spore infection under natural conditions (the picture of natural infected silkworm was quoted from http://cs.gxcy.gov.cn).
(TIF)

Figure S2 **Observation of silkworms at different infection time points.** C: Uninfected silkworms. Exp 1: silkworms fed with 5×10^4^ spores per larvae. Exp 2: silkworms fed with 1×10^5^ spores per larvae. Exp 3: silkworm fed with 3×10^5^ spores per larvae. d: days post-infection.
(TIF)

Figure S3 ***N. bombycis*** **spores in different tissues of severely infected silkworms at day 10.** (A∼F) Observation of *N. bombycis* spores in the midgut, fat body, silk gland, hemolymph, malpighian tubule and ovary, respectively.
(TIF)

Figure S4 **Phylogenetic analysis and domain prediction of HPXs(A) and BmSPNs (B).** The phylogenetic tree was reconstructed using MEGA 4.0 with 1,500-times bootstrap sampling and domains were predicted by pfam software. The up-regulated genes were indicated by the arrows pointing upward. The arrows pointing downward showed the down-regulated genes.
(TIF)

Table S1 **Gene specific primers for real-time quantitative PCR.**
(DOC)

Table S2 **Ratios and annotation of all** ***N. bombycis*** **induced genes.**
(XLS)

Table S3 **Genes involved in the 14 major clusters of induced expression profile analysis and the corresponding log_2_ value of their fold change.**
(XLS)

Table S4 **The ratios of genes mentioned in this report.**
(DOC)

Table S5 ***N. bombycis*** **induced enzymes involved in general metabolism of silkworm by KEGG prediction.**
(DOC)

Table S6 **The number of differential expression genes in basic metabolism pathways.**
(DOC)

Table S7 **Gene specific primers for real-time quantitative PCR in Toll and JAK/STAT pathways in silkworm.**
(DOC)

Table S8 **Genes related to** ***N. bombycis*** **induced silkworm cellular immune response.**
(DOC)

Table S9 **Ratio of silkworm immune-related genes induced by different pathogens.**
(XLS)
