# Supplementary figures and images for: Genome-Wide Transcriptional Response of Silkworm (Bombyx mori) to Infection by the Microsporidian Nosema bombycis
Source: PLoS One. 2013 Dec 30;8(12):e84137. doi: 10.1371/journal.pone.0084137 (PMC3875524; doi:10.1371/journal.pone.0084137)

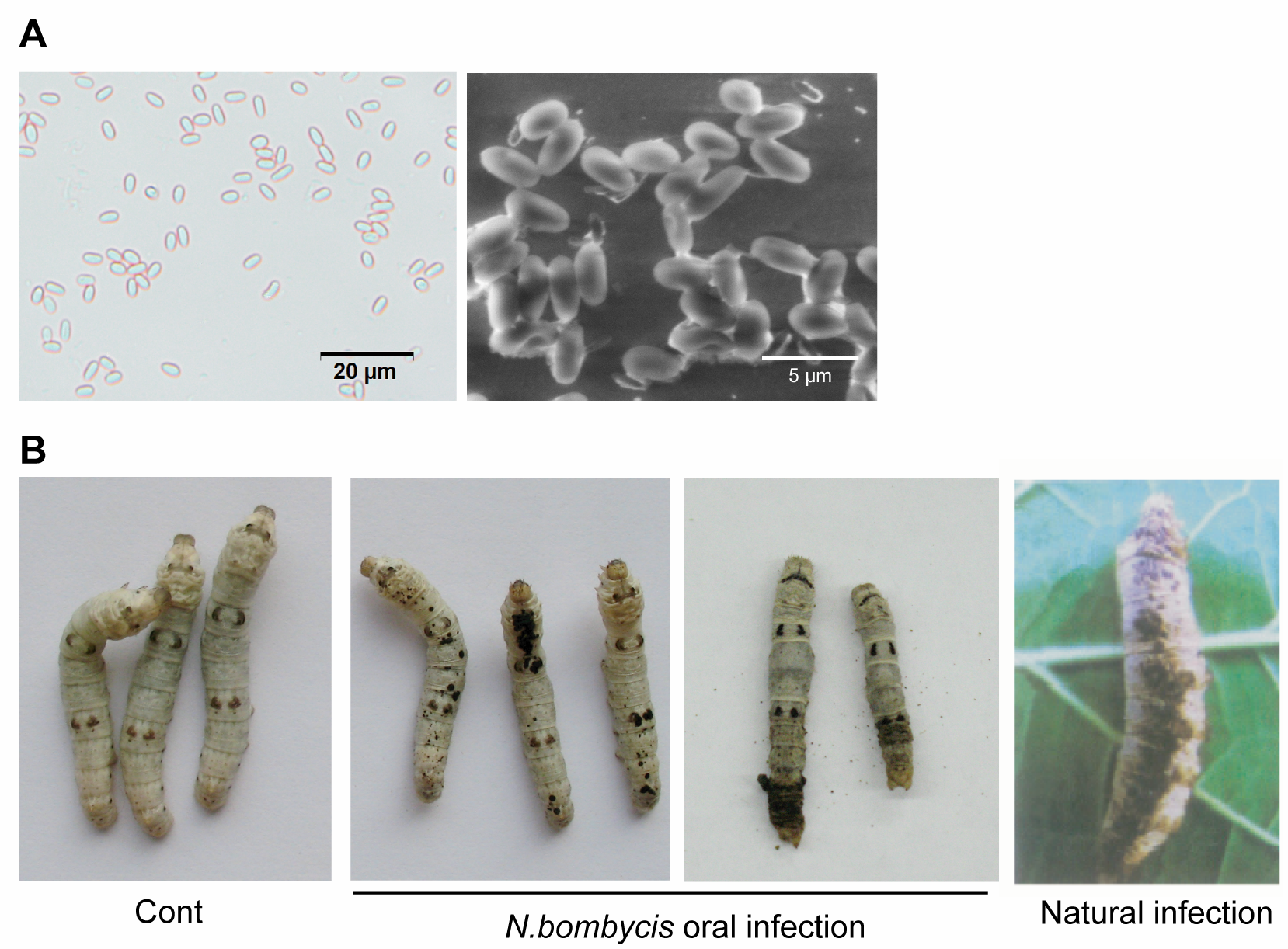

Supplement: Figure S1 — Observation of purified spores and N. bombycis oral-infected silkworms. (A) N. bombycis spores as visualized by optical and electron microscopes. (B) Oral infection with isolated N. bombycis causes severe pébrine, which is similar to natural infection. Cont:Uninfected silkworm larvae. N. bombycis infected silkworm larvae exhibited prickly ash spots on the cuticles and N. bombycis-infected silkworms molted with difficultly. The disease symptoms were similar to spore infection under natural conditions (the picture of natural infected silkworm was quoted from http://cs.gxcy.gov.cn). (TIF) [file pone.0084137.s001.tif]

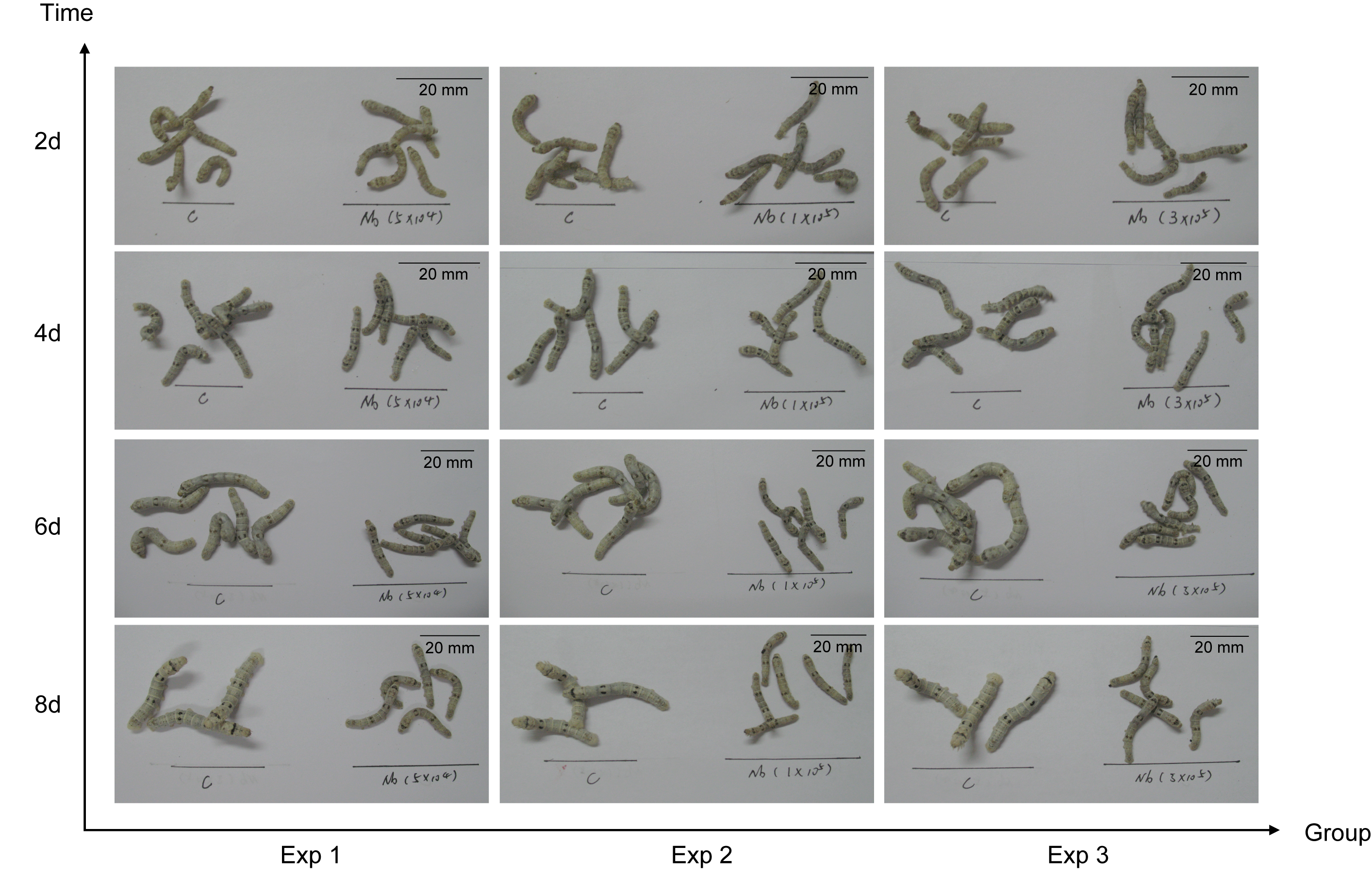

Supplement: Figure S2 — Observation of silkworms at different infection time points. C: Uninfected silkworms. Exp 1: silkworms fed with 5×10^4^ spores per larvae. Exp 2: silkworms fed with 1×10^5^ spores per larvae. Exp 3: silkworm fed with 3×10^5^ spores per larvae. d: days post-infection. (TIF) [file pone.0084137.s002.tif]

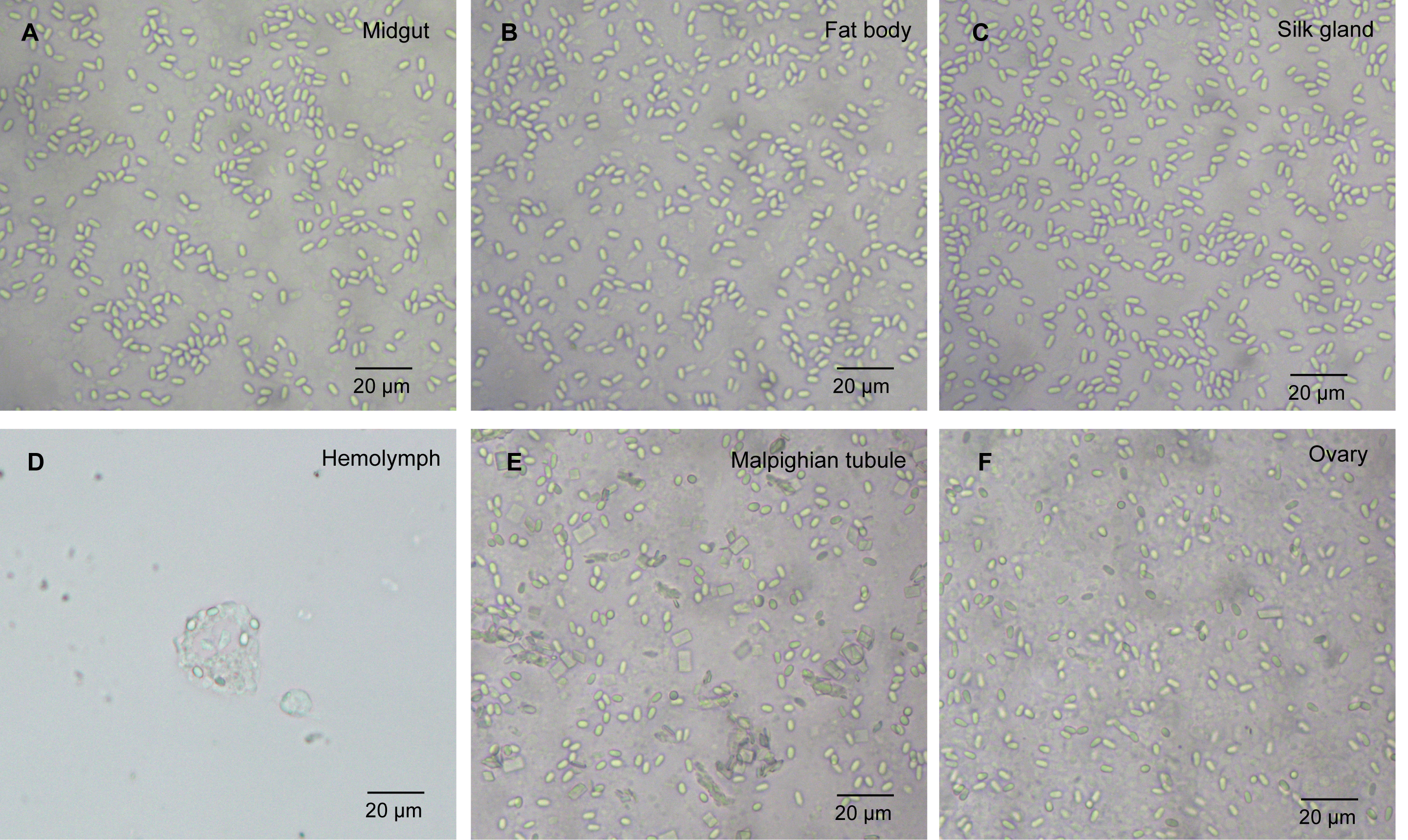

Supplement: Figure S3 — N. bombycis spores in different tissues of severely infected silkworms at day 10. (A∼F) Observation of N. bombycis spores in the midgut, fat body, silk gland, hemolymph, malpighian tubule and ovary, respectively. (TIF) [file pone.0084137.s003.tif]

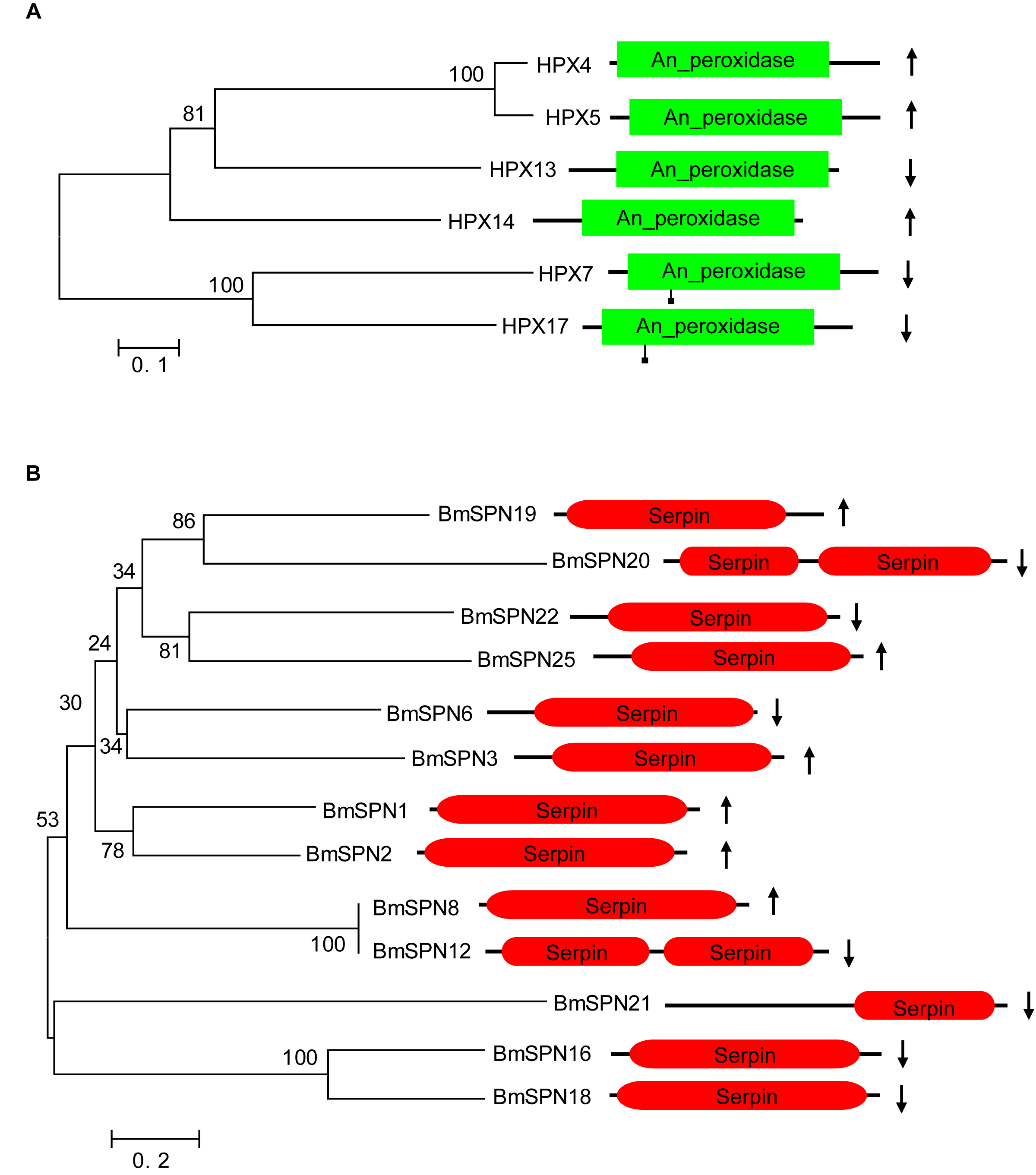

Supplement: Figure S4 — Phylogenetic analysis and domain prediction of HPXs(A) and BmSPNs (B). The phylogenetic tree was reconstructed using MEGA 4.0 with 1,500-times bootstrap sampling and domains were predicted by pfam software. The up-regulated genes were indicated by the arrows pointing upward. The arrows pointing downward showed the down-regulated genes. (TIF) [file pone.0084137.s004.tif]
