# Supplementary material for: Genome-Wide Transcriptional Response of Silkworm (Bombyx mori) to Infection by the Microsporidian Nosema bombycis
Source: PLoS One. 2013 Dec 30;8(12):e84137. doi: 10.1371/journal.pone.0084137 (PMC3875524; doi:10.1371/journal.pone.0084137)
Supplement: Table S1 — Gene specific primers for real-time quantitative PCR. (DOC) [file pone.0084137.s005.doc]

| **Table S1** | | | | |
| --- | --- | --- | --- | --- |
| **Gene specific primers for real-time quantitative PCR** | | | | |
| Gene ID | Probe ID | Primer name | Forward primer | Reverse primer |
| BGIBMGA012864 | sw22599 | PGRP-S3 | ACTATTCCTTCCCCTTCG | ATGGGCACTGGACTGACT |
| BGIBMGA012865 | sw17703 | PGRP-S4 | GACAGCGTCATCACCCCT | TCTCTCACAGTTTGCCCG |
| BGIBMGA011609 | sw10605 | β-GRP2 | TCAGCGGCAGAATCACATCA | CGCACGCTATCTTCACTACGC |
| BGIBMGA001590 | sw01191 | OTC | ATTACGACCTCGCTTGTGACG | TTCATGGTCAGAGCGATCCTT |
| BGIBMGA004735 | sw22015 | SPN12 | TCTCGCTGTAACATCCGTGC | TGGAAAGTCCTCTTCACCTCAG |
| BGIBMGA006623 | sw22902 | CTL 11 | CCACAGAGGAGATACACAAGTTCA | CGTAATGTGCAACCAGCCTT |
| BGIBMGA014360 | sw04645 | DEP | ATGAAGGTGTACGCGTGCTTGT | TCCGTCCTCGAGCGAGAAAGTC |
| BGIBMGA010938 | [sw19962](http://silkworm.swu.edu.cn/microarray/Bmarray.php?search=yes&range=sw19962) | ADC | GAACTTACAGAGTCGGTTTGGTCA | AGCCATAGACCAGACTGAACAAGT |
| BGIBMGA008824 | sw18399 | ALP | CGCATCACGACAACTACGC | ACGTGGGTGTGGTCAGCAGT |
