# Supplementary material for: Genome-Wide Transcriptional Response of Silkworm (Bombyx mori) to Infection by the Microsporidian Nosema bombycis
Source: PLoS One. 2013 Dec 30;8(12):e84137. doi: 10.1371/journal.pone.0084137 (PMC3875524; doi:10.1371/journal.pone.0084137)
Supplement: Table S4 — The ratios of genes mentioned in this report. (DOC) [file pone.0084137.s008.doc]

| **Table S4** | | | | | |
| --- | --- | --- | --- | --- | --- |
| **The ratios of genes mentioned in this report** | | | | | |
| Probe/Gene ID | Gene | 2 dpi | 4 dpi | 6 dpi | 8 dpi |
| **Genes involved in Juvenile hormone of silkworm** | | | | | |
| sw22915 | AcAT | 1.4394 | 1.2159 | 4.302367 | 1.5326 |
| sw20582 | HMGS | 0.9922 | 0.8593 | 0.238633 | 0.4563 |
| sw09011 | FPPS | 1 | 1 | 1 | 0.4695 |
| sw05537 | FAMet | 1.1898 | 0.9452 | 2.286467 | 0.9075 |
| sw08195 | FAMet | 0.937433 | 2.0817 | 1.1343 | 2.655 |
| sw14489 | JHAMT | 1.502967 | 2.9991 | 5.190633 | 11.7367 |
| sw22960 | JHAMT | 0.8375 | 1.5372 | 11.80783 | 12.2754 |
| sw18135 | JHAMT | 0.954867 | 1.4342 | 18.9694 | 12.8921 |
| sw01578 | AS | 1 | 1 | 1 | 2.2818 |
| sw01366 | AS | 0.9634 | 1.4284 | 1.646333 | 3.1315 |
| sw14035 | JHE | 1 | 1 | 1 | 0.1795 |
| sw20366 | JHEH | 1 | 1 | 1 | 0.1001 |
| sw14267 | JHEH | 1.806833 | 0.8047 | 3.351967 | 0.9135 |
| sw02554 | JHEH | 1 | 1 | 1 | 0.3298 |
| sw08871 | JHBP | 0.820333 | 0.8256 | 0.817333 | 2.7277 |
| sw06424 | JHBP | 1 | 1.4794 | 16.36497 | 16.9176 |
| sw00529 | JHBP | 0.984033 | 1.5261 | 2.486267 | 2.8331 |
| sw14879 | JHBP | 1.774667 | 1.6451 | 6.789233 | 1 |
| sw20455 | JHBP | 0.847 | 2.3936 | 0.190133 | 0.3556 |
| sw13985 | JHBP | 1.141433 | 0.6554 | 0.6083 | 3.4222 |
| sw11688 | JHDK | 6.853233 | 0.6554 | 1.316 | 0.7734 |
| sw04517 | JHDK | 0.7067 | 0.6812 | 0.9484 | 2.2528 |
| sw11434 | EcR | 0.990433 | 0.6161 | 0.2359 | 1 |
| **Genes involved in Toll pathway** | | | | | |
| sw10605 | βGRP2 | 1.243867 | 1.3182 | 0.7322 | 3.1389 |
| sw20413 | βGRP4 | 1.536 | 2.2322 | 4.3038 | 5.721 |
| sw22599 | PGRP-S3 | 1 | 0.9528 | 1.006133 | 17.5859 |
| sw17703 | PGRP-S4 | 2.2579 | 0.9624 | 0.8544 | 18.7991 |
| sw20945 | BmCLIP7 | 1 | 1.1434 | 1 | 2.427 |
| sw22653 | BmCLIP12 | 1 | 1.2923 | 1 | 2.2868 |
| sw20515 | BmCLIP15 | 1 | 1 | 1 | 0.0366 |
| sw16610 | BmSpz2 | 2.441467 | 0.7716 | 0.107567 | 0.0922 |
| sw05360 | Toll-10 | 1.1 | 1.11 | 0.95 | 2.52 |
| BGIBMGA013866 | Gloverin 4-like | 5.358133 | 2.9471 | 5.871467 | 16.6843 |
| BGIBMGA013865 | Gloverin A1 | 7.069433 | 1 | 9.0105 | 1 |
| sw22043 | Gloverin A5 | 3.5001 | 1.7356 | 1.4172 | 2.6712 |
| sw12523 | Gloverin A6 | 4.228233 | 3.0304 | 1.312133 | 2.4926 |
| BGIBMGA013864 | Gloverin A4 | 4.086733 | 1 | 1.904033 | 1 |
| Sw11372 | Cecropin B | 1.031267 | 1 | 0.032567 | 0.0732 |
| BGIBMGA000017 | Cecropin D1 | 2.269333 | 1 | 0.392167 | 1 |
| sw15815 | Moricin I | 0.7317 | 3.6581 | 0.1319 | 1.4544 |
| sw09761 | Moricin like-C2 | 1 | 4.289 | 4.695833 | 3.2698 |
| sw17805 | Attacin B | 2.991367 | 1.7358 | 0.286933 | 0.9453 |
| sw08844 | Lebocin 3 | 2.161667 | 1.6208 | 2.892467 | 4.3776 |
| sw11016 | Moricin like-B6 | 0.815267 | 0.8306 | 4.127367 | 2.0624 |
| **Genes involved in silkworm serine protease cascade melanization pathway** | | | | | |
| sw03832 | BmCTL4 | 1.6231 | 1 | 0.035467 | 0.022 |
| sw15689 | BmCTL5 | 1.609567 | 0.7849 | 1.685067 | 0.1088 |
| sw15509 | BmCTL7 | 0.853733 | 0.582 | 0.104333 | 0.6766 |
| sw09190 | BmCTL9 | 0.8105 | 0.8739 | 0.182767 | 0.4371 |
| sw22902 | BmCTL11 | 0.926233 | 5.7761 | 10.74393 | 17.3349 |
| sw00143 | BmCTL15 | 1.169967 | 1 | 0.0598 | 1 |
| sw07855 | BmCTL16 | 1.679 | 0.6283 | 0.373167 | 0.0342 |
| sw08273 | BmCTL18 | 1 | 1 | 1 | 0.0838 |
| sw11925 | BmSPN1 | 0.902667 | 1.3462 | 1.945033 | 3.5359 |
| sw18472 | BmSPN2 | 1.191167 | 1.3722 | 1.344667 | 2.1283 |
| BGIBMGA010212 | BmSPN3 | 1 | 1.4703 | 1 | 2.0677 |
| sw16003 | BmSPN6 | 1.030333 | 1 | 0.156467 | 1 |
| sw19598 | BmSPN8 | 0.707933 | 0.4896 | 0.431533 | 5.7162 |
| sw22015 | BmSPN12 | 1.1029 | 0.4873 | 0.083533 | 0.0397 |
| sw22273 | BmSPN12 | 1.0028 | 0.6514 | 0.050767 | 0.0562 |
| sw13114 | BmSPN16 | 0.901567 | 1 | 0.032833 | 0.068 |
| sw19259 | BmSPN18 | 0.923333 | 1 | 0.044567 | 0.2169 |
| sw16168 | BmSPN19 | 0.798967 | 1.962 | 2.972833 | 3.3472 |
| sw06085 | BmSPN20 | 0.793 | 1.6997 | 0.0663 | 0.6119 |
| sw11465 | BmSPN21 | 1.336467 | 1 | 0.063733 | 1 |
| BGIBMGA013848 | BmSPN22 | 1 | 1 | 1 | 0.2036 |
| sw16035 | BmSPN25 | 0.914233 | 0.9218 | 1.516533 | 2.0268 |
| sw04645 | BmDEP | 1 | 3.9513 | 2.9837 | 11.555 |
| sw20014 | BmDD | 1 | 1.1266 | 1 | 0.4897 |
| sw13482 | BmTH | 1.9998 | 1.4547 | 2.038533 | 0.0571 |
| sw15390 | BmPPAE2 | 0.8451 | 1.3213 | 0.6398 | 0.1159 |
| sw20327 | BmPPAE | 1.1792 | 1.1694 | 2.132167 | 2.1213 |
| sw21973 | BmPPO1 | 1.122633 | 1 | 0.4551 | 1 |
