# Supplementary material for: Genome-Wide Transcriptional Response of Silkworm (Bombyx mori) to Infection by the Microsporidian Nosema bombycis
Source: PLoS One. 2013 Dec 30;8(12):e84137. doi: 10.1371/journal.pone.0084137 (PMC3875524; doi:10.1371/journal.pone.0084137)
Supplement: Table S5 — N. bombycis induced enzymes involved in general metabolism of silkworm by KEGG prediction. (DOC) [file pone.0084137.s009.doc]

| **Table S5** | | | | | | |
| --- | --- | --- | --- | --- | --- | --- |
| ***Nb* induced enzymes involved in general metabolism of silkworm by KEGG prediction** | | | | | | |
| **Enzyme ID** | **Enzyme** | **Probe ID** | **2d** | **4d** | **6d** | **8d** |
| Pyrimidine metabolism | | | | | | |
| ec:1.17.4.1 | ribonucleoside-diphosphate reductase | sw07356 | 1.260967 | 1.3273 | 1.739233 | 2.9337 |
| ec:1.17.4.1 | ribonucleoside-diphosphate reductase | sw04795 | 1.286167 | 2.4175 | 1.941567 | 5.4899 |
| ec:1.3.5.2 | dihydroorotate dehydrogenase (quinone) | sw18510 | 0.941333 | 0.8225 | 1.893933 | 4.678 |
| ec:2.7.7.8 | polyribonucleotide nucleotidyltransferase | sw02311 | 0.9713 | 0.9608 | 1.047967 | 0.4887 |
| ec:2.7.7.7 | DNA-directed DNA polymerase | sw04751 | 1.083033 | 1.1159 | 1.1233 | 2.3782 |
| ec:2.7.4.9 | dTMP kinase | sw15988 | 1.163633 | 0.7772 | 2.092767 | 1 |
| ec:2.1.1.45 | thymidylate synthase | sw16054 | 1.1111 | 1.2706 | 1.159233 | 2.719 |
| ec:3.5.4.5 | cytidine deaminase | sw00005 | 1 | 0.4099 | 1 | 1 |
| Purine metabolism | | | | | | |
| ec:1.17.4.1 | ribonucleoside-diphosphate reductase | sw07356 | 1.260967 | 1.3273 | 1.739233 | 2.9337 |
| ec:1.17.4.1 | ribonucleoside-diphosphate reductase | sw04795 | 1.286167 | 2.4175 | 1.941567 | 5.4899 |
| ec:3.5.4.10 | IMP cyclohydrolase | sw18903 | 0.780433 | 0.6511 | 0.371833 | 2.0157 |
| ec:3.6.1.15 | nucleoside-triphosphatase | sw01593 | 1.001033 | 1.7438 | 1.776567 | 2.3215 |
| ec:3.6.1.15 | nucleoside-triphosphatase | sw03876 | 1.0113 | 1 | 1 | 0.3649 |
| ec:3.6.1.15 | nucleoside-triphosphatase | sw13968 | 1.044833 | 1 | 1 | 0.4796 |
| ec:3.6.1.15 | nucleoside-triphosphatase | sw07877 | 0.8152 | 0.9409 | 0.249267 | 0.9424 |
| ec:3.6.1.15 | nucleoside-triphosphatase | sw14545 | 0.967467 | 0.519 | 0.376367 | 1.3814 |
| ec:3.6.1.15 | nucleoside-triphosphatase | sw09644 | 1.511267 | 0.9358 | 2.7651 | 0.7086 |
| ec:2.7.7.8 | polyribonucleotide nucleotidyltransferase | sw02311 | 0.9713 | 0.9608 | 1.047967 | 0.4887 |
| ec:2.7.7.7 | DNA-directed DNA polymerase | sw04751 | 1.083033 | 1.1159 | 1.1233 | 2.3782 |
| ec:4.6.1.1 | adenylate cyclase | sw11800 | 1 | 1.2606 | 4.353933 | 2.0875 |
| ec:2.7.6.1 | ribose-phosphate diphosphokinase | sw04154 | 1 | 0.2634 | 0.486467 | 5.6242 |
| ec:2.7.6.1 | ribose-phosphate diphosphokinase | sw09898 | 0.946567 | 0.6974 | 0.473433 | 0.9033 |
| ec:6.3.4.4 | adenylosuccinate synthase | sw09546 | 1 | 1 | 1 | 0.3664 |
| ec:3.1.4.17 | 3',5'-cyclic-nucleotide phosphodiesterase | sw10070 | 1 | 1 | 1 | 0.32 |
| ec:3.1.4.17 | 3',5'-cyclic-nucleotide phosphodiesterase | sw19272 | 1 | 1 | 1 | 0.0588 |
| ec:3.6.1.3 | adenosinetriphosphatase | sw00464 | 0.831833 | 1 | 0.285467 | 1 |
| ec:3.6.1.3 | adenosinetriphosphatase | sw13178 | 1 | 1.3238 | 1 | 0.4522 |
| ec:3.6.1.3 | adenosinetriphosphatase | sw06368 | 0.8985 | 1 | 0.193333 | 1 |
| ec:3.6.1.3 | adenosinetriphosphatase | sw08321 | 1.147 | 1.5287 | 2.3843 | 2.1686 |
| ec:6.3.2.6 | phosphoribosylaminoimidazolesuccinocarboxamide synthase | sw17399 | 1.048733 | 0.9713 | 3.7469 | 2.8137 |
| ec:3.5.4.4 | adenosine deaminase | sw09901 | 1.167733 | 0.4183 | 1.142467 | 0.5983 |
| ec:2.1.2.3 | phosphoribosylaminoimidazolecarboxamide formyltransferase | sw18903 | 0.780433 | 0.6511 | 0.371833 | 2.0157 |
| Phenylalanine metabolism | | | | | | |
| ec:1.13.11.27 | 4-hydroxyphenylpyruvate dioxygenase | sw07607 | 0.8617 | 1.515 | 3.597733 | 4.0982 |
| ec:1.11.1.7 | peroxidase | sw02487 | 1.127467 | 1 | 0.130067 | 0.1167 |
| ec:1.11.1.7 | peroxidase | sw20228 | 0.5807 | 1 | 0.043267 | 0.0378 |
| ec:4.1.1.28 | aromatic-L-amino-acid decarboxylase | sw15376 | 1.406633 | 1 | 0.0175 | 1 |
| ec:2.6.1.1 | aspartate transaminase | sw10674 | 0.866133 | 0.8394 | 1.048033 | 2.2529 |
| ec:1.14.16.1 | phenylalanine 4-monooxygenase | sw01062 | 1 | 3.9413 | 6.349833 | 1.7111 |
| Tyrosine metabolism | | | | | | |
| ec:1.1.1.1 | alcohol dehydrogenase | sw13775 | 1.293667 | 1.4678 | 2.014033 | 5.2166 |
| ec:1.13.11.27 | 4-hydroxyphenylpyruvate dioxygenase | sw07607 | 0.8617 | 1.515 | 3.597733 | 4.0982 |
| ec:3.7.1.2 | fumarylacetoacetase | sw01333 | 0.850233 | 1.1484 | 1.296667 | 3.7656 |
| ec:4.1.1.28 | aromatic-L-amino-acid decarboxylase | sw15376 | 1.406633 | 1 | 0.0175 | 1 |
| ec:5.3.3.12 | L-dopachrome isomerase | sw17419 | 1 | 1.5898 | 2.137933 | 1.039 |
| ec:1.14.18.1 | monophenol monooxygenase | sw21973 | 1.122633 | 1 | 0.4551 | 1 |
| ec:2.6.1.1 | aspartate transaminase | sw10674 | 0.866133 | 0.8394 | 1.048033 | 2.2529 |
| ec:1.13.11.5 | homogentisate 1,2-dioxygenase | sw08054 | 1 | 2.2566 | 2.762567 | 3.8841 |
| ec:1.14.16.2 | tyrosine 3-monooxygenase | sw13482 | 1.9998 | 1.4547 | 2.038533 | 0.0571 |
| Tryptophan metabolism | | | | | | |
| ec:1.13.11.11 | tryptophan 2,3-dioxygenase | sw00689 | 1.005433 | 1.6053 | 1.769467 | 2.566 |
| ec:1.11.1.6 | catalase | sw03619 | 0.991967 | 1.0664 | 2.416167 | 3.0291 |
| ec:2.3.1.9 | acetyl-CoA C-acetyltransferase | sw22915 | 1.4394 | 1.2159 | 4.302367 | 1.5326 |
| ec:3.7.1.3 | kynureninase | sw14459 | 1 | 1.3423 | 3.773233 | 1.2952 |
| ec:1.2.4.2 | oxoglutarate dehydrogenase (succinyl-transferring) | sw17970 | 0.8942 | 1.2089 | 1.0291 | 2.0348 |
| ec:1.2.4.2 | oxoglutarate dehydrogenase (succinyl-transferring) | sw10755 | 0.8557 | 1.3291 | 2.4044 | 4.1587 |
| ec:4.1.1.28 | aromatic-L-amino-acid decarboxylase | sw15376 | 1.406633 | 1 | 0.0175 | 1 |
| Arginine and proline metabolism | | | | | | |
| ec:2.7.2.11 | glutamate 5-kinase | sw09946 | 0.971533 | 1 | 0.103533 | 1 |
| ec:1.5.1.2 | pyrroline-5-carboxylate reductase | sw00432 | 0.8712 | 0.9251 | 0.408233 | 1 |
| ec:6.3.4.5 | argininosuccinate synthase | sw15269 | 1.112433 | 1.7369 | 1.147767 | 2.4994 |
| ec:1.14.13.39 | nitric-oxide synthase | sw20485 | 1 | 1 | 1 | 0.1934 |
| ec:1.14.13.39 | nitric-oxide synthase | sw20844 | 1.1711 | 2.4564 | 2.855633 | 2.0916 |
| ec:6.3.1.2 | glutamate1-ammonia ligase | sw21168 | 1 | 2.8358 | 4.1138 | 0.3253 |
| ec:2.6.1.1 | aspartate transaminase | sw10674 | 0.866133 | 0.8394 | 1.048033 | 2.2529 |
| Valine, leucine and isoleucine degradation | | | | | | |
| ec:1.3.99.3 | acyl-CoA dehydrogenase | sw14804 | 1.324033 | 0.9979 | 0.488367 | 1.0423 |
| ec:2.3.1.168 | dihydrolipoyllysine-residue (2-methylpropanoyl)transferase | sw07108 | 1.1209 | 0.8938 | 2.442467 | 2.2332 |
| ec:2.3.1.9 | acetyl-CoA C-acetyltransferase | sw22915 | 1.4394 | 1.2159 | 4.302367 | 1.5326 |
| ec:2.8.3.5 | 3-oxoacid CoA-transferase | sw18740 | 0.920833 | 0.9302 | 1.411633 | 2.0011 |
| ec:2.6.1.42 | branched-chain-amino-acid transaminase | sw08509 | 1 | 1.1813 | 2.335633 | 2.9761 |
| ec:2.3.3.10 | hydroxymethylglutaryl-CoA synthase | sw20582 | 0.9922 | 0.8593 | 0.238633 | 0.4563 |
| ec:1.2.1.27 | methylmalonate-semialdehyde dehydrogenase (acylating) | sw11744 | 0.880733 | 0.8298 | 1.658067 | 2.5752 |
| Lysine degradation | | | | | | |
| ec:2.3.1.9 | acetyl-CoA C-acetyltransferase | sw22915 | 1.4394 | 1.2159 | 4.302367 | 1.5326 |
| ec:1.2.4.2 | oxoglutarate dehydrogenase (succinyl-transferring) | sw17970 | 0.8942 | 1.2089 | 1.0291 | 2.0348 |
| ec:1.2.4.2 | oxoglutarate dehydrogenase (succinyl-transferring) | sw10755 | 0.8557 | 1.3291 | 2.4044 | 4.1587 |
| Cysteine and methionine metabolism | | | | | | |
| ec:2.5.1.6 | methionine adenosyltransferase | sw22876 | 1.111467 | 1.4331 | 4.646533 | 3.1692 |
| ec:4.4.1.14 | 1-aminocyclopropane-1-carboxylate synthase | sw12156 | 0.998467 | 1.3263 | 1.667967 | 2.0285 |
| ec:2.1.1.37 | DNA (cytosine-5-)-methyltransferase | sw15948 | 0.972267 | 1.2208 | 1.397 | 3.558 |
| ec:2.6.1.1 | aspartate transaminase | sw10674 | 0.866133 | 0.8394 | 1.048033 | 2.2529 |
| Alanine, aspartate and glutamate metabolism | | | | | | |
| ec:6.3.5.4 | asparagine synthase (glutamine-hydrolysing) | sw11016 | 0.815267 | 0.8306 | 4.127367 | 2.0624 |
| ec:6.3.4.5 | argininosuccinate synthase | sw15269 | 1.112433 | 1.7369 | 1.147767 | 2.4994 |
| ec:6.3.4.4 | adenylosuccinate synthase | sw09546 | 1 | 1 | 1 | 0.3664 |
| ec:6.3.1.2 | glutamate1-ammonia ligase | sw21168 | 1 | 2.8358 | 4.1138 | 0.3253 |
| ec:2.6.1.1 | aspartate transaminase | sw10674 | 0.866133 | 0.8394 | 1.048033 | 2.2529 |
| Citrate cycle (TCA cycle) | | | | | | |
| ec:2.3.3.1 | citrate (Si)-synthase | sw17936 | 1.036967 | 1.0268 | 2.0826 | 1.3956 |
| ec:1.2.4.2 | oxoglutarate dehydrogenase (succinyl-transferring) | sw17970 | 0.8942 | 1.2089 | 1.0291 | 2.0348 |
| ec:1.2.4.2 | oxoglutarate dehydrogenase (succinyl-transferring) | sw10755 | 0.8557 | 1.3291 | 2.4044 | 4.1587 |
| Pentose phosphate pathway | | | | | | |
| ec:4.1.2.13 | fructose-bisphosphate aldolase | sw06691 | 0.906933 | 0.6125 | 0.370367 | 0.797 |
| ec:2.7.6.1 | ribose-phosphate diphosphokinase | sw04154 | 1 | 0.2634 | 0.486467 | 5.6242 |
| ec:2.7.6.1 | ribose-phosphate diphosphokinase | sw09898 | 0.946567 | 0.6974 | 0.473433 | 0.9033 |
| ec:3.1.3.11 | fructose-bisphosphatase | sw04448 | 1.053733 | 0.5803 | 0.422033 | 0.541 |
| Butanoate metabolism | | | | | | |
| ec:2.3.1.9 | acetyl-CoA C-acetyltransferase | sw22915 | 1.4394 | 1.2159 | 4.302367 | 1.5326 |
| ec:2.8.3.5 | 3-oxoacid CoA-transferase | sw18740 | 0.920833 | 0.9302 | 1.411633 | 2.0011 |
| ec:2.3.3.10 | hydroxymethylglutaryl-CoA synthase | sw20582 | 0.9922 | 0.8593 | 0.238633 | 0.4563 |
| Pyruvate metabolism | | | | | | |
| ec:2.3.1.9 | acetyl-CoA C-acetyltransferase | sw22915 | 1.4394 | 1.2159 | 4.302367 | 1.5326 |
| ec:3.6.1.7 | acylphosphatase | sw04586 | 1 | 1 | 1 | 0.4927 |
| ec:6.2.1.1 | acetate1-CoA ligase | sw10630 | 1 | 1 | 1 | 0.249 |
| Pantothenate and CoA biosynthesis | | | | | | |
| ec:4.1.1.11 | aspartate 1-decarboxylase | sw07783 | 1 | 1 | 1 | 0.0745 |
| ec:2.6.1.42 | branched-chain-amino-acid transaminase | sw08509 | 1 | 1.1813 | 2.335633 | 2.9761 |
| One carbon pool by folate | | | | | | |
| ec:1.5.1.6 | formyltetrahydrofolate dehydrogenase | sw18442 | 1.3384 | 2.6738 | 1.3056 | 16.1345 |
| ec:1.5.1.5 | methylenetetrahydrofolate dehydrogenase (NADP+) | sw11215 | 1.198667 | 0.9394 | 3.325233 | 2.4786 |
| ec:2.1.1.45 | thymidylate synthase | sw16054 | 1.1111 | 1.2706 | 1.159233 | 2.719 |
| ec:2.1.2.3 | phosphoribosylaminoimidazolecarboxamide formyltransferase | sw18903 | 0.780433 | 0.6511 | 0.371833 | 2.0157 |
| Thiamine (Vitamin B1) metabolism | | | | | | |
| ec:3.6.1.15 | nucleoside-triphosphatase | sw01593 | 1.001033 | 1.7438 | 1.776567 | 2.3215 |
| ec:3.6.1.15 | nucleoside-triphosphatase | sw03876 | 1.0113 | 1 | 1 | 0.3649 |
| ec:3.6.1.15 | nucleoside-triphosphatase | sw13968 | 1.044833 | 1 | 1 | 0.4796 |
| ec:3.6.1.15 | nucleoside-triphosphatase | sw07877 | 0.8152 | 0.9409 | 0.249267 | 0.9424 |
| ec:3.6.1.15 | nucleoside-triphosphatase | sw14545 | 0.967467 | 0.519 | 0.376367 | 1.3814 |
| ec:3.6.1.15 | nucleoside-triphosphatase | sw09644 | 1.511267 | 0.9358 | 2.7651 | 0.7086 |
| Drug metabolism | | | | | | |
| ec:3.5.4.5 | cytidine deaminase | sw00005 | 1 | 0.4099 | 1 | 1 |
| ec:1.1.1.1 | alcohol dehydrogenase | sw13775 | 1.293667 | 1.4678 | 2.014033 | 5.2166 |
| ec:1.14.13.8 | flavin-containing monooxygenase | sw18680 | 3.0656 | 1 | 0.060133 | 0.0719 |
| ec:1.14.13.8 | flavin-containing monooxygenase | sw13424 | 1 | 1 | 1 | 4.3936 |
| ec:2.5.1.18 | glutathione transferase | sw14925 | 1.2368 | 1.477 | 2.8052 | 1.398 |
| ec:2.5.1.18 | glutathione transferase | sw02205 | 1.000333 | 1 | 0.3545 | 1 |
| ec:2.5.1.18 | glutathione transferase | sw09921 | 1.104667 | 1.413 | 3.622567 | 1.245 |
| ec:2.5.1.18 | glutathione transferase | sw17370 | 1.019167 | 0.7309 | 0.858 | 2.5188 |
| ec:2.5.1.18 | glutathione transferase | sw18220 | 0.786367 | 0.9039 | 2.3669 | 8.3084 |
| ec:2.5.1.18 | glutathione transferase | sw10371 | 0.901833 | 1.0678 | 0.483233 | 1.0012 |
| Styrene degradation | | | | | | |
| ec:3.7.1.2 | fumarylacetoacetase | sw01333 | 0.850233 | 1.1484 | 1.296667 | 3.7656 |
| ec:1.13.11.5 | homogentisate 1,2-dioxygenase | sw08054 | 1 | 2.2566 | 2.762567 | 3.8841 |
| Metabolism of xenobiotics by cytochrome P450 | | | | | | |
| ec:1.1.1.1 | alcohol dehydrogenase | sw13775 | 1.293667 | 1.4678 | 2.014033 | 5.2166 |
| ec:2.5.1.18 | glutathione transferase | sw14925 | 1.2368 | 1.477 | 2.8052 | 1.398 |
| ec:2.5.1.18 | glutathione transferase | sw02205 | 1.000333 | 1 | 0.3545 | 1 |
| ec:2.5.1.18 | glutathione transferase | sw09921 | 1.104667 | 1.413 | 3.622567 | 1.245 |
| ec:2.5.1.18 | glutathione transferase | sw17370 | 1.019167 | 0.7309 | 0.858 | 2.5188 |
| ec:2.5.1.18 | glutathione transferase | sw18220 | 0.786367 | 0.9039 | 2.3669 | 8.3084 |
| ec:2.5.1.18 | glutathione transferase | sw10371 | 0.901833 | 1.0678 | 0.483233 | 1.0012 |
| Isoquinoline alkaloid biosynthesis | | | | | | |
| ec:4.1.1.28 | aromatic-L-amino-acid decarboxylase | sw15376 | 1.406633 | 1 | 0.0175 | 1 |
| ec:1.14.18.1 | monophenol monooxygenase | sw21973 | 1.122633 | 1 | 0.4551 | 1 |
| ec:2.6.1.1 | aspartate transaminase | sw10674 | 0.866133 | 0.8394 | 1.048033 | 2.2529 |
| ec:1.14.16.2 | tyrosine 3-monooxygenase | sw13482 | 1.9998 | 1.4547 | 2.038533 | 0.0571 |
| Methane metabolism | | | | | | |
| ec:1.14.13.8 | flavin-containing monooxygenase | sw18680 | 3.0656 | 1 | 0.060133 | 0.0719 |
| ec:1.14.13.8 | flavin-containing monooxygenase | sw13424 | 1 | 1 | 1 | 4.3936 |
| ec:4.1.2.13 | fructose-bisphosphate aldolase | sw06691 | 0.906933 | 0.6125 | 0.370367 | 0.797 |
| ec:4.2.1.11 | phosphopyruvate hydratase | sw19290 | 0.773533 | 0.6977 | 0.4991 | 1.8375 |
| ec:2.6.1.52 | phosphoserine transaminase | sw07324 | 0.874167 | 0.8474 | 1.1189 | 2.6231 |
| ec:1.11.1.7 | peroxidase | sw02487 | 1.127467 | 1 | 0.130067 | 0.1167 |
| ec:1.11.1.7 | peroxidase | sw20228 | 0.5807 | 1 | 0.043267 | 0.0378 |
| ec:1.11.1.6 | catalase | sw03619 | 0.991967 | 1.0664 | 2.416167 | 3.0291 |
| ec:3.1.3.11 | fructose-bisphosphatase | sw04448 | 1.053733 | 0.5803 | 0.422033 | 0.541 |
| ec:6.2.1.1 | acetate1-CoA ligase | sw10630 | 1 | 1 | 1 | 0.249 |
| Glutathione metabolism | | | | | | |
| ec:1.17.4.1 | ribonucleoside-diphosphate reductase | sw16645 | 1 | 1.2298 | 1 | 3.6311 |
| ec:1.17.4.1 | ribonucleoside-diphosphate reductase | sw04795 | 1.286167 | 2.4175 | 1.941567 | 5.4899 |
| ec:2.3.2.2 | gamma-glutamyltransferase | sw02154 | 1.130667 | 0.7164 | 1.0713 | 2.9355 |
| ec:2.5.1.18 | glutathione transferase | sw14925 | 1.2368 | 1.477 | 2.8052 | 1.398 |
| ec:2.5.1.18 | glutathione transferase | sw02205 | 1.000333 | 1 | 0.3545 | 1 |
| ec:2.5.1.18 | glutathione transferase | sw09921 | 1.104667 | 1.413 | 3.622567 | 1.245 |
| ec:2.5.1.18 | glutathione transferase | sw17370 | 1.019167 | 0.7309 | 0.858 | 2.5188 |
| ec:2.5.1.18 | glutathione transferase | sw18220 | 0.786367 | 0.9039 | 2.3669 | 8.3084 |
| ec:2.5.1.18 | glutathione transferase | sw10371 | 0.901833 | 1.0678 | 0.483233 | 1.0012 |
| Nitrogen metabolism | | | | | | |
| ec:6.3.5.4 | asparagine synthase (glutamine-hydrolysing) | sw11016 | 0.815267 | 0.8306 | 4.127367 | 2.0624 |
| ec:6.3.1.2 | glutamate1-ammonia ligase | sw21168 | 1 | 2.8358 | 4.1138 | 0.3253 |
| Glycerolipid metabolism | | | | | | |
| ec:3.1.1.3 | triacylglycerol lipase | sw19707 | 0.8041 | 1 | 0.117367 | 0.52 |
| ec:3.1.1.3 | triacylglycerol lipase | sw18197 | 1.6664 | 2.7879 | 1.893633 | 5.9043 |
| ec:2.3.1.51 | 1-acylglycerol-3-phosphate O-acyltransferase | sw12087 | 1.025733 | 1.3588 | 1.6144 | 0.4628 |
| Synthesis and degradation of ketone bodies | | | | | | |
| ec:2.3.1.9 | acetyl-CoA C-acetyltransferase | sw22915 | 1.4394 | 1.2159 | 4.302367 | 1.5326 |
| ec:2.8.3.5 | 3-oxoacid CoA-transferase | sw18740 | 0.920833 | 0.9302 | 1.411633 | 2.0011 |
| ec:2.3.3.10 | hydroxymethylglutaryl-CoA synthase | sw20582 | 0.9922 | 0.8593 | 0.238633 | 0.4563 |
| Fatty acid metabolism | | | | | | |
| ec:1.1.1.1 | alcohol dehydrogenase | sw13775 | 1.293667 | 1.4678 | 2.014033 | 5.2166 |
| ec:1.3.99.3 | acyl-CoA dehydrogenase | sw14804 | 1.324033 | 0.9979 | 0.488367 | 1.0423 |
| ec:2.3.1.9 | acetyl-CoA C-acetyltransferase | sw22915 | 1.4394 | 1.2159 | 4.302367 | 1.5326 |
| ec:1.3.3.6 | acyl-CoA oxidase | sw14804 | 1.324033 | 0.9979 | 0.488367 | 1.0423 |
| Glycerophospholipid metabolism | | | | | | |
| ec:3.1.4.46 | glycerophosphodiester phosphodiesterase | sw16854 | 1 | 1.5621 | 5.508233 | 1.4548 |
| ec:3.1.1.4 | phospholipase A2 | sw02938 | 1.3525 | 1.3126 | 0.1218 | 0.2317 |
| ec:2.3.1.51 | 1-acylglycerol-3-phosphate O-acyltransferase | sw12087 | 1.025733 | 1.3588 | 1.6144 | 0.4628 |
| ec:1.1.5.3 | glycerol-3-phosphate dehydrogenase | sw15541 | 0.725133 | 0.709 | 0.820133 | 0.3368 |
| Alpha-Linolenic acid metabolism | | | | | | |
| ec:1.3.3.6 | acyl-CoA oxidase | sw14804 | 1.324033 | 0.9979 | 0.488367 | 1.0423 |
| ec:3.1.1.4 | phospholipase A2 | sw02938 | 1.3525 | 1.3126 | 0.1218 | 0.2317 |
| Arachidonic acid metabolism | | | | | | |
| ec:3.3.2.10 | soluble epoxide hydrolase | sw02554 | 1 | 1 | 1 | 0.3298 |
| ec:3.3.2.10 | soluble epoxide hydrolase | sw14267 | 1.806833 | 0.8047 | 3.351967 | 0.9135 |
| ec:2.3.2.2 | gamma-glutamyltransferase | sw02154 | 1.130667 | 0.7164 | 1.0713 | 2.9355 |
| ec:3.1.1.4 | phospholipase A2 | sw02938 | 1.3525 | 1.3126 | 0.1218 | 0.2317 |
| Sphingolipid metabolism | | | | | | |
| ec:3.2.1.23 | beta-galactosidase | sw05025 | 1.089967 | 1.6126 | 5.4347 | 4.2474 |
| ec:3.1.4.12 | sphingomyelin phosphodiesterase | sw06076 | 0.939167 | 1 | 0.132333 | 1.2371 |
| Amino sugar and nucleotide sugar metabolism | | | | | | |
| ec:3.2.1.52 | beta-N-acetylhexosaminidase | sw19182 | 0.909067 | 1 | 0.1379 | 1 |
| ec:3.2.1.52 | beta-N-acetylhexosaminidase | sw20215 | 1.503067 | 3.1193 | 1.017933 | 7.053 |
| ec:1.1.1.22 | UDP-glucose 6-dehydrogenase | sw13973 | 0.9185 | 1 | 0.048567 | 1 |
| ec:2.4.1.16 | chitin synthase | sw08657 | 0.9853 | 0.968 | 0.329 | 0.1825 |
| ec:2.7.1.1 | hexokinase | sw08786 | 1 | 1 | 1 | 0.3036 |
| ec:3.2.1.14 | chitinase | sw10122 | 1 | 1 | 9.008933 | 7.237 |
| ec:3.2.1.14 | chitinase | sw09267 | 0.9846 | 1 | 0.067167 | 1 |
| ec:3.2.1.14 | chitinase | sw19054 | 0.603567 | 1 | 0.166367 | 0.1132 |
| ec:3.2.1.14 | chitinase | sw08485 | 0.7658 | 1 | 0.076767 | 0.2841 |
| Glycolysis/Gluconeogenesis | | | | | | |
| ec:1.1.1.1 | alcohol dehydrogenase | sw13775 | 1.293667 | 1.4678 | 2.014033 | 5.2166 |
| ec:4.1.2.13 | fructose-bisphosphate aldolase | sw06691 | 0.906933 | 0.6125 | 0.370367 | 0.797 |
| ec:4.2.1.11 | phosphopyruvate hydratase | sw19290 | 0.773533 | 0.6977 | 0.4991 | 1.8375 |
| ec:3.1.3.11 | fructose-bisphosphatase | sw04448 | 1.053733 | 0.5803 | 0.422033 | 0.541 |
| ec:2.7.1.1 | hexokinase | sw08786 | 1 | 1 | 1 | 0.3036 |
| ec:5.3.1.1 | triose-phosphate isomerase | sw11392 | 0.834533 | 0.6827 | 0.4176 | 1.0859 |
| ec:6.2.1.1 | acetate1-CoA ligase | sw10630 | 1 | 1 | 1 | 0.249 |
| Starch and sucrose metabolism | | | | | | |
| ec:1.1.1.22 | UDP-glucose 6-dehydrogenase | sw13973 | 0.9185 | 1 | 0.048567 | 1 |
| ec:3.2.1.28 | alpha,alpha-trehalase | sw09196 | 1.1174 | 1.3 | 2.2672 | 0.7383 |
| ec:3.2.1.26 | beta-fructofuranosidase | sw02518 | 0.7522 | 1.5035 | 9.148933 | 2.7201 |
| ec:2.7.1.1 | hexokinase | sw08786 | 1 | 1 | 1 | 0.3036 |
| Galactose metabolism | | | | | | |
| ec:3.2.1.26 | beta-fructofuranosidase | sw02518 | 0.7522 | 1.5035 | 9.148933 | 2.7201 |
| ec:3.2.1.23 | beta-galactosidase | sw05025 | 1.089967 | 1.6126 | 5.4347 | 4.2474 |
| ec:2.7.1.1 | hexokinase | sw08786 | 1 | 1 | 1 | 0.3036 |
| Fructose and mannose metabolism | | | | | | |
| ec:4.1.2.13 | fructose-bisphosphate aldolase | sw06691 | 0.906933 | 0.6125 | 0.370367 | 0.797 |
| ec:1.1.1.14 | L-iditol 2-dehydrogenase | sw22553 | 1 | 0.569 | 0.843667 | 3.9335 |
| ec:3.1.3.11 | fructose-bisphosphatase | sw04448 | 1.053733 | 0.5803 | 0.422033 | 0.541 |
| ec:2.7.1.1 | hexokinase | sw08786 | 1 | 1 | 1 | 0.3036 |
| ec:5.3.1.1 | triose-phosphate isomerase | sw11392 | 0.834533 | 0.6827 | 0.4176 | 1.0859 |
| Various types of N-glycan biosynthesis | | | | | | |
| ec:3.2.1.52 | beta-N-acetylhexosaminidase | sw19182 | 0.909067 | 1 | 0.1379 | 1 |
| ec:3.2.1.52 | beta-N-acetylhexosaminidase | sw20215 | 1.503067 | 3.1193 | 1.017933 | 7.053 |
| ec:3.2.1.113 | mannosyl-oligosaccharide 1,2-alpha-mannosidase | sw18931 | 1 | 2.6383 | 4.4973 | 15.1379 |
| Glycosaminoglycan degradation | | | | | | |
| ec:3.2.1.52 | beta-N-acetylhexosaminidase | sw19182 | 0.909067 | 1 | 0.1379 | 1 |
| ec:3.2.1.23 | beta-galactosidase | sw05025 | 1.089967 | 1.6126 | 5.4347 | 4.2474 |
| ec:3.2.1.52 | beta-N-acetylhexosaminidase | sw20215 | 1.503067 | 3.1193 | 1.017933 | 7.053 |
| Basic transcription factors | | | | | | |
|  | transcription factor ap-1 | sw16467 | 1.1745 | 2.618 | 1.409467 | 1.7484 |
|  | transcription factor collier- partial | sw08764 | 1.1217 | 1.0328 | 1.486533 | 2.8022 |
|  | transcription factor e74 | sw00164 | 1 | 1 | 1 | 0.3024 |
|  | transcription factor ets | sw11166 | 0.798967 | 3.0826 | 2.232033 | 0.4519 |
|  | transcription initiation factor alpha subunit | sw11096 | 1.0844 | 1.3728 | 1.269533 | 2.3454 |
|  | transcription initiation factor tfiid subunit 7 | sw10844 | 1 | 1 | 1 | 2.064 |
|  | transcriptional adapter 1-like | sw14129 | 1.049467 | 2.1828 | 1.312133 | 2.7446 |
|  | forkfead transcription factor G1 | sw19390 | 1 | 1.5178 | 3.504833 | 3.0495 |
