# Supplementary material for: Genome-Wide Transcriptional Response of Silkworm (Bombyx mori) to Infection by the Microsporidian Nosema bombycis
Source: PLoS One. 2013 Dec 30;8(12):e84137. doi: 10.1371/journal.pone.0084137 (PMC3875524; doi:10.1371/journal.pone.0084137)
Supplement: Table S6 — The number of differential expression genes in basic metabolism pathways. (DOC) [file pone.0084137.s010.doc]

| **Table S6** | | | | | | | | | |
| --- | --- | --- | --- | --- | --- | --- | --- | --- | --- |
| **The number of differential expression genes in basic metabolism pathways** | | | | | | | | | |
| Basic metabolism | Involved pathway | 2 dpi | | 4 dpi | | 6 dpi | | 8 dpi | |
|  |  | Up^#^ | Down^*^ | Up^#^ | Down^*^ | Up^#^ | Down^*^ | Up^#^ | Down^*^ |
| Nucleotide metabolism |  |  |  |  |  |  |  |  |  |
|  | Pyrimidine metabolism | / | / | 1 | 1 | 1 | / | 5 | 1 |
|  | purine metabolism | / | / | 1 | 2 | 4 | 8 | 10 | 7 |
| Amino acid metabolism |  |  |  |  |  |  |  |  |  |
|  | Phenylalanine metabolism | / | / | 1 | / | 2 | 3 | 3 | 2 |
|  | Tyrosine metabolism | / | / | 1 | / | 5 | 2 | 5 | 1 |
|  | Tryptophan metabolism | / | / | / | / | 4 | 1 | 4 | / |
|  | Arginine and proline metabolism | / | / | 2 | / | 2 | 2 | 3 | 2 |
|  | Valine, leucine and isoleucine degradation | / | / | / | / | 3 | 2 | 5 | 1 |
|  | Lysine degradation | / | / | / | / | 2 | / | 2 | / |
|  | Cysteine and methionine metabolism | / | / | / | / | 1 | / | 5 | 1 |
|  | Alanine, aspartate and Glutamate metabolism | / | / | 2 | / | 3 | / | 4 | 3 |
| Carbohydrate metabolism |  |  |  |  |  |  |  |  |  |
|  | Citrate cycle (TCA cycle) | / | / | / | / | 2 | / | 2 | / |
|  | Pentose phosphate pathway | / | / | / | 1 | / | 4 | 1 | / |
|  | Butanoate metabolism | / | / | / | / | 1 | 1 | 1 | 1 |
|  | Pyruvate metabolism | / | / | / | / | 1 | / | / | 2 |
| Cofactor and Vitamin metabolism |  |  |  |  |  |  |  |  |  |
|  | Pantothenate and CoA biosynthesis | / | / | / | / | 1 | / | 1 | 1 |
|  | One carbon pool by folate | / | / | 1 | / | 1 | 1 | 4 | / |
|  | Thiamine (Vitamin B1) metabolism | / | / | / | / | 1 | 2 | 1 | 2 |
| Xenobiotic biotransformation |  |  |  |  |  |  |  |  |  |
|  | Drug metabolism | 1 | / | / | 1 | 4 | 3 | 4 | 1 |
|  | Styrene degradation | / | / | 2 | / | 2 | / | 3 | / |
|  | Metabolism of xenobiotics by cytochrome P450 | / | / | / | / | 4 | 2 | 3 | / |
|  | Isoquinoline alkaloid biosynthesis | / | / | / | / | 1 | 2 | 1 | 1 |
|  | Methane metabolism | 1 | / | / | / | 1 | 6 | 3 | 4 |
|  | Glutathione metabolism | / | / | 1 | / | 3 | 2 | 5 | / |
|  | Nitrogen metabolism | / | / | 1 | / | 2 | / | 1 | 1 |
| Genetic information processing |  |  |  |  |  |  |  |  |  |
|  | Basic transcrition factors | / | / | 3 | / | 2 | / | 5 | 2 |
| Lipid metabolim |  |  |  |  |  |  |  |  |  |
|  | Glycerolipid metabolism | / | / | 1 | / | / | 1 | 1 | 1 |
|  | Synthesis and degradation of ketone bodies | / | / | / | / | 1 | 1 | 1 | 1 |
|  | Fatty acid metabolism | / | / | / | / | 2 | 1 | 1 | / |
|  | Glycerophospholipid metabolism | / | / | / | / | 1 | 1 | / | 3 |
|  | Alpha-Linolenic acid metabolism | / | / | / | / | / | 2 | / | 1 |
|  | Arachidonic acid metabolism | / | / | / | / | 1 | 1 | 1 | 2 |
|  | Sphingolipid metabolism | / | / | / | / | 1 | 1 | 1 | / |
| Saccharometabolism and glucoprotein |  |  |  |  |  |  |  |  |  |
|  | Amino sugar and nucleotide sugar metabolism | / | / | 1 | 1 | 1 | 6 | 2 | 4 |
|  | Glycolysis/ Gluconeogenesis | / | / | / | / | 1 | 4 | 1 | 2 |
|  | Starch and sucrose metabolism | / | / | / | / | 2 | 1 | 1 | 1 |
|  | Galactose metabolism | / | / | / | / | 2 | / | 2 | 1 |
|  | Fructose and mannose metabolism | / | / | / | / | / | 3 | 1 | 1 |
|  | Various types of N-glycan biosynthesis | / | / | 2 | / | 1 | 1 | 2 | / |
|  | Glycosaminoglycan degradation | / | / | 1 | / | 1 | 1 | 2 | / |

^#^ The number of up-regulated genes involved in metabolism pathways

^*^ The number of down-regulated genes involved in metabolism pathways
