# Supplementary material for: Genome-Wide Transcriptional Response of Silkworm (Bombyx mori) to Infection by the Microsporidian Nosema bombycis
Source: PLoS One. 2013 Dec 30;8(12):e84137. doi: 10.1371/journal.pone.0084137 (PMC3875524; doi:10.1371/journal.pone.0084137)
Supplement: Table S7 — Gene specific primers for real-time quantitative PCR in Toll and JAK/STAT pathways in silkworm. (DOC) [file pone.0084137.s011.doc]

| **Table S7** | | | | |
| --- | --- | --- | --- | --- |
| **Gene specific primers for real-time quantitative PCR in Toll and JAK/STAT pathways in silkworm** | | | | |
| Gene ID | Probe ID | Primer name | Forward primer | Reverse primer |
| BGIBMGA002397 | sw08256 | Spz1 | AGGATTCGCCTCACAGTCAC | ATTTTCAGTTCGGGATGCTT |
| BGIBMGA002869 | sw05796 | Myd88 | TAATAGACTCGGAGGAATG | CGATACTAATAGCCTGTGC |
| BGIBMGA002494 | sw20628 | Tube | ATTCTATTGATGGACCGACGC | CTCCATTTACTTCAATCGGCTT |
| BGIBMGA000063 | sw07930 | Pelle | AATAAAAGAGAAGGCTCGTCA | TGTGTAGGAGTCTATGGAACC |
| BGIBMGA011037 | sw08028 | Toll-1 | CACTTCGGCTGGACTCTGATG | AATCAACAAGACCTGGAACGG |
| BGIBMGA008840 | sw11928 | Toll-9 | CGTTGCGATGCCTGATGAC | TCTCCTGAAATTCCGCCCTA |
| BGIBMGA011082 | sw05360 | Toll-10 | CGGTTTGTCAAGTTTACGC | GGAGGCAAGGCAGTTAGAG |
| BGIBMGA011025 | sw09777 | Toll-11 | TACCATTCACCCGTCTATC | TATCTGGCATAACGAATCTAA |
| BGIBMGA005642 | sw14077 | Dome | TTACACTGGCATTGAACACC | TGAATCACTAAGCACATCGG |
| BGIBMGA001739 | sw15699 | STAT1 | TGAGGCATTGTTTGGCGT | CTCCTGGGGGGCGTGACT |
| BGIBMGA004082 | sw00739 | Hop | TGTGGCAAAACGGCAGTGAGA | CAGGATGGTGCGGATGAAAAG |
