# Supplementary material for: Genome-Wide Transcriptional Response of Silkworm (Bombyx mori) to Infection by the Microsporidian Nosema bombycis
Source: PLoS One. 2013 Dec 30;8(12):e84137. doi: 10.1371/journal.pone.0084137 (PMC3875524; doi:10.1371/journal.pone.0084137)
Supplement: Table S8 — Genes related to N. bombycis induced silkworm cellular immune response. (DOC) [file pone.0084137.s012.doc]

| **Table S8** | | | | | | |
| --- | --- | --- | --- | --- | --- | --- |
| **Genes related to *N.bombycis* induced silkworm cellular immune response** | | | | | | |
| **Name** | **Probe ID** | **Gene ID** | **Ratio** | | | |
|  |  |  | **2d** | **4d** | **6d** | **8d** |
| BmCTL4 | sw03832 | BGIBMGA005948-TA | 1.6231 | 1 | 0.035467 | 0.022 |
| BmCTL5 | sw15689 | BGIBMGA005977-TA | 1.609567 | 0.7849 | 1.685067 | 0.1088 |
| BmCTL7 | sw15509 | BGIBMGA003660-TA | 0.853733 | 0.582 | 0.104333 | 0.6766 |
| BmCTL9 | sw09190 | BGIBMGA012488-TA | 0.8105 | 0.8739 | 0.182767 | 0.4371 |
| BmCTL11 | sw22902 | BGIBMGA006623-TA | 0.926233 | 5.7761 | 10.74393 | 17.3349 |
| BmCTL15 | sw00143 | BGIBMGA002163-TA | 1.169967 | 1 | 0.0598 | 1 |
| BmCTL16 | sw07855 | BGIBMGA007553-TA | 1.679 | 0.6283 | 0.373167 | 0.0342 |
| BmCTL18 | sw08273 | BGIBMGA008247-TA | 1 | 1 | 1 | 0.0838 |
| CASP3 | sw22502 | BGIBMGA006131-TA | 1 | 3.582 | 5.102 | 3.3297 |
| CAT1 | sw03619 | BGIBMGA000701-TA | 0.991967 | 1.0664 | 2.416167 | 3.0291 |
| cSPH13 | sw22653 | BGIBMGA014436-TA | 1 | 1.2923 | 1 | 2.2868 |
| FREP3 | sw15340 | BGIBMGA012306-TA | 1 | 1 | 1 | 0.2904 |
| Hemolin | sw07814 | BGIBMGA008736-TA | 1 | 1 | 1 | 0.1777 |
| HPX4 | sw21485 | BGIBMGA000238-TA | 1 | 1.2747 | 9.771533 | 1.6673 |
| HPX5 | sw17863 | BGIBMGA000457-TA | 1 | 1.2931 | 12.09797 | 2.0996 |
| HPX7 | sw20228 | BGIBMGA005680-TA | 0.5807 | 1 | 0.043267 | 0.0378 |
| HPX13 | sw08597 | BGIBMGA012739-TA | 1.032067 | 1 | 0.074633 | 0.0374 |
| HPX14 | sw20315 | BGIBMGA012740-TA | 1 | 1.1397 | 4.9229 | 0.8779 |
| HPX17 | sw02487 | BGIBMGA014559-TA | 1.127467 | 1 | 0.130067 | 0.1167 |
| IG2 | sw02946 | BGIBMGA000622-TA | 0.917467 | 1.2491 | 2.3393 | 1.4761 |
| IG6 | sw20465 | BGIBMGA002409-TA | 1 | 1 | 1 | 0.1659 |
| IG8 | sw09094 | BGIBMGA004546-TA | 0.748133 | 1.0805 | 2.460633 | 1.4351 |
| IG9 | sw01099 | BGIBMGA004547-TA | 0.726933 | 0.8329 | 2.065967 | 1.4051 |
| IRP1 | sw04276 | BGIBMGA013991-TA | 1 | 4.1482 | 16.0263 | 0.5417 |
| IRP2 | sw04645 | BGIBMGA014360-TA | 1 | 3.9513 | 2.9837 | 11.555 |
| Jafrac | sw09911 | BGIBMGA002406-TA | 1.099967 | 0.9038 | 0.457067 | 1.2191 |
| LYS1 | sw00728 | BGIBMGA012264-TA | 1 | 1.3801 | 4.000133 | 2.8117 |
| LYS2 | sw13847 | BGIBMGA010439-TA | 2.191433 | 2.268 | 0.467667 | 2.1552 |
| LYS3 | sw01851 | BGIBMGA014001-TA | 0.929467 | 1.1003 | 1.4004 | 2.2158 |
| ML2 | sw00887 | BGIBMGA008221-TA | 0.773933 | 0.9873 | 0.5651 | 3.021 |
| NPC2 | sw11619 | BGIBMGA008405-TA | 1.056533 | 0.7419 | 0.285933 | 0.4671 |
| RIP1 | sw02310 | BGIBMGA005381-TA | 0.872933 | 0.8012 | 0.075667 | 1.1 |
| SOCS1 | sw07854 | BGIBMGA009204-TA | 1 | 1.5769 | 1 | 2.9789 |
| SOD3 | sw00814 | BGIBMGA002798-TA | 1 | 1.6085 | 1 | 0.1697 |
| SOD4 | sw14555 | BGIBMGA002907-TA | 0.8487 | 1.4703 | 3.593067 | 0.6047 |
| SCRASP4 | sw05844 | BGIBMGA003679-TA | 0.857967 | 1 | 0.09 | 0.235 |
| SCRB9 | sw22236 | BGIBMGA011408-TA | 1 | 1 | 1 | 2.2343 |
